# Total Synthesis and Stereochemical Assignment of Alternapyrone

**DOI:** 10.3390/molecules30071597

**Published:** 2025-04-03

**Authors:** Hui Zhang, Jiaxuan Feng, Di Wang, Bencan Tang, Chao Xu, Tao Ye

**Affiliations:** 1 State Key Laboratory of Chemical Oncogenomics, Peking University Shenzhen Graduate School, Shenzhen 518055, China; huizhang@stu.pku.edu.cn (H.Z.); jxfeng@stu.pku.edu.cn (J.F.); 2 Department of Chemical and Environmental Engineering, Faculty of Science and Engineering, The University of Nottingham Ningbo China, Ningbo 315100, China; di.wang@nottingham.edu.cn (D.W.); bencan.tang@nottingham.edu.cn (B.T.)

**Keywords:** total synthesis, natural products, absolute configuration, prediction

## Abstract

Alternapyrone, a bioactive polyketide produced by the fungal host *Aspergillus oryzae*, is biosynthesized by a polyketide synthase encoded by the *alt1-5* gene cluster. Despite its known bioactivity, the stereochemical configuration of the three stereogenic centers in its polyketide backbone has remained unresolved. In this study, we determined the complete stereostructure of alternapyrone using an integrative approach that combines predictive, rule-based stereochemical analysis with experimental validation through total synthesis. The efficient total synthesis enabled the precise assignment of the hypothesized stereochemistry by matching the synthetic product to the natural compound. This comprehensive study conclusively established the absolute configuration of alternapyrone.

## 1. Introduction

**Figure 1:**
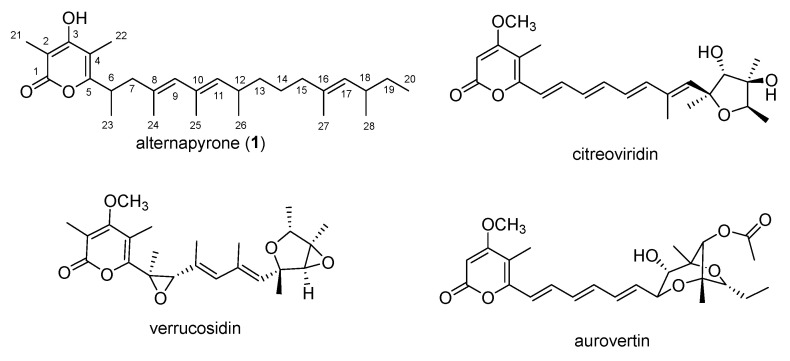
Representative fungal metabolites with α-pyrone.

Fungi have been the source of many groundbreaking drugs, playing a critical role in the treatment of chronic conditions. These fungal-derived compounds have proven invaluable in managing chronic infections, autoimmune diseases, and high cholesterol. Recently, metabolites derived from fungi have entered clinical trials, showing promise in treating chronic diseases such as cancer and drug-resistant depression. Fungi-derived natural products often include polyketides, a large and diverse group of secondary metabolites known for their structural variety [1]. Among these, polyketides featuring an α-pyrone structure with a 6-alkenyl chain display diverse methylation patterns, leading to various metabolites such as citreoviridin [2], verrucosidin [3], and aurovertin B [4] (Figure 1). The carbon backbone of these polyketides is biosynthesized by polyketide synthases (PKSs), a class of enzymes with catalytic domains resembling those of mammalian fatty acid synthases. Fungal PKSs are classified as iterative type I PKSs [5]. During the assembly of polyketide-derived backbones, specific auxiliary domains within fungal PKSs participate selectively in particular condensation cycles, introducing functional diversity into the polyketide chain. In collaboration with our research partners, we have developed a unified stereochemical model that elucidates the configuration of complex polyhydroxylated polyketides. This work has led to the formulation of a biochemistry-based rule, enabling the prediction of the absolute configuration of fungal-derived reduced polyketides [6].

In 2005, Isao Fujii and colleagues introduced the *alt1-5* genes, originally cloned from the fungal pathogen *Alternaria solani*, into the fungal host *Aspergillus oryzae*. These genes encode a polyketide synthase responsible for producing a polyketide compound called alternapyrone [7]. Since then, researchers have continued to explore methods for synthesizing alternapyrone analogs [8,9]. Using high-resolution electron ionization mass spectrometry (EI-MS), ^13^C NMR, ^1^H NMR, and two-dimensional NMR techniques, the two-dimensional structure of alternapyrone was elucidated, and a biosynthetic pathway for its catalytic production was proposed. In 2018, Yit-Heng Chooi’s team tested the biological activity of alternapyrone. Their findings showed that alternapyrone exhibited cytotoxicity against mouse myeloma cells, with a minimum inhibitory concentration (MIC) of 3.1 μg/mL. Notably, its activity against neonatal foreskin fibroblast non-tumor cells was eight times lower (MIC = 25 μg/mL), suggesting a degree of specificity for tumor cells [8]. In 2022, Wattana-Amorn and colleagues investigated the role of C-methylation in alternapyrone biosynthesis. Their work provided insights into the methylation programming of highly reducing polyketide synthases (HR-PKSs) [10]. We have long been interested in the stereochemistry of polyketides, particularly in developing reliable methods to resolve the stereochemical complexity of natural products. To this end, we integrated established stereochemical principles with insights into the biosynthetic pathway to propose the absolute stereochemistry of alternapyrone. This study outlines our efforts to achieve the total synthesis of alternapyrone and confirm its proposed stereochemistry.

**Figure 2:**
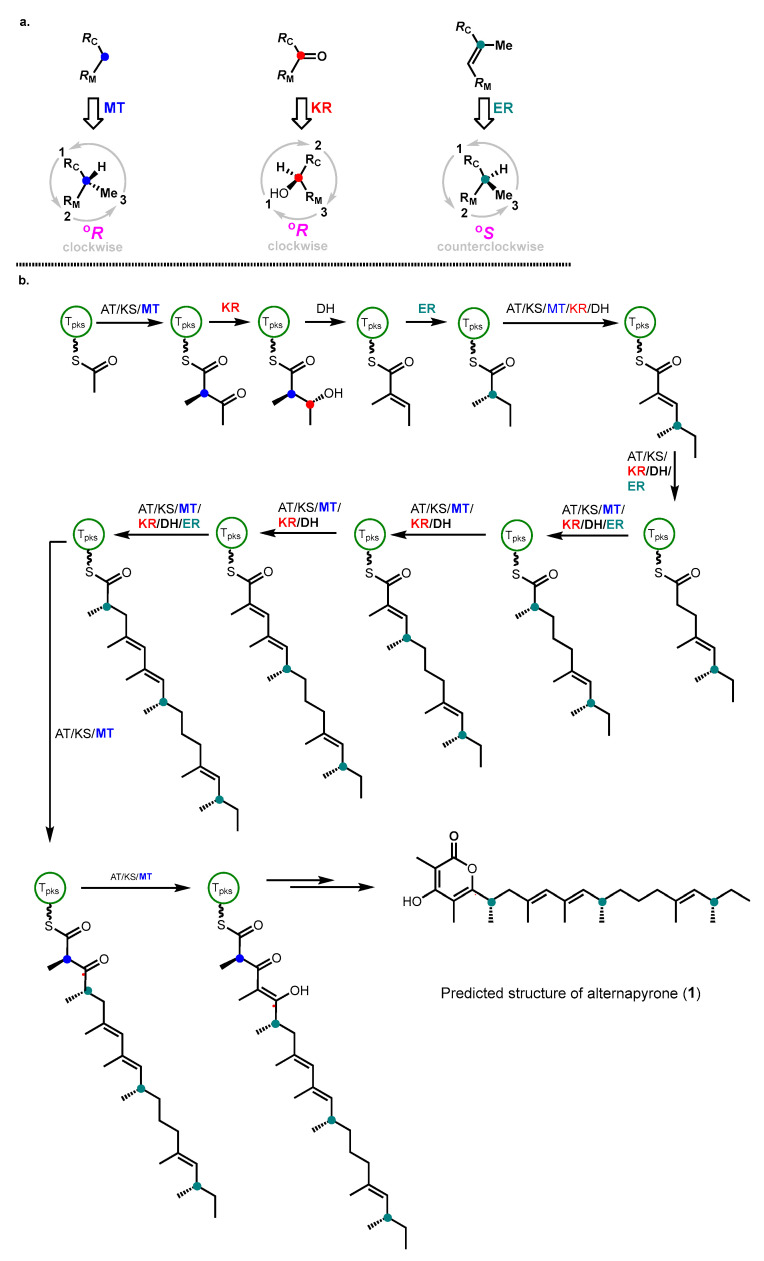
(**a**) Definition of *°R*/*°S* configuration. (**b**) Biochemistry-based rule guided prediction of the structure of alternapyrone. AT: Acyltransferase, MT: Methyltransferase, KR: Keto reductase, ER: Enoyl reductase, KS: Keto synthase, DH: Dehydratase.

The biochemistry-based rule offers a promising approach to simplifying the stereochemical determination of fungal-derived polyketide compounds [6]. We propose that this rule could be applied to predict the absolute configuration of fungal-reduced alternapyrone, potentially optimizing synthetic processes and minimizing redundant efforts. Fungal HR-PKSs (highly reducing polyketide synthases) execute a series of complex reactions, including initiation, elongation, methylation, reduction, and dehydration. According to the “Biochemistry-guided Rule” [6], the keto reductase (KR) domain reduces keto groups to hydroxyl groups with a *°R*-configuration, the enoyl reductase (ER) domain reduces enoyl groups to methyl groups with a *°S*-configuration, and the methyltransferase (MT) domain incorporates methyl groups with a *°R*-configuration (Figure 2a). It is worth noting that the *°R/°S* configurations described by the biochemistry-based rule differ slightly from the conventional R/S stereochemical notation. The *°R*-configuration is determined using the following steps: identify and label the group on the polyketide chain elongation side as RC; label the group on the chain initiation side as RM; assign priority to the groups in this order: OH > RC > RM > Me > H; view the stereogenic center from the side opposite to the hydrogen atom (the group with the lowest priority) and arrange the remaining three groups in descending order of priority. If the sequence follows a clockwise pattern, it is designated as a *°R* configuration. If the sequence is counterclockwise, it is classified as a *°S* configuration. This systematic approach enhances clarity and provides a robust framework for stereochemical prediction of the absolute configuration of fungal-derived polyketides.

The absolute configuration of alternapyrone was predicted based on the rule mentioned above and is illustrated in Figure 2b. Specifically, the enoyl reductase (ER) domain sequentially reduces the intermediate, resulting in an °*S*-configuration for the three methyl groups. To validate this stereochemical prediction, it is crucial to synthesize the compound. Experimental verification will confirm the proposed structure and offer valuable insights into the reliability of our biochemistry-based prediction model, supporting its application to other fungal polyketide systems.

**Scheme 1:**
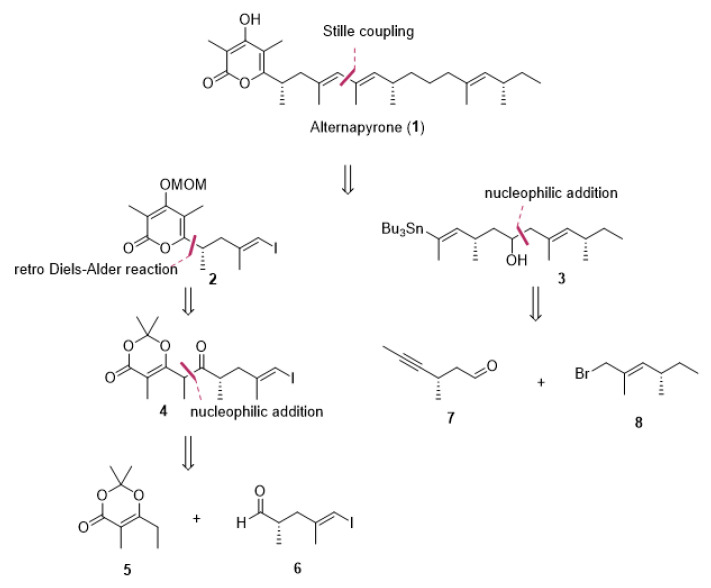
Retrosynthetic analysis of alternapyrone (**1**).

The retrosynthetic analysis of alternapyrone (**1**) is outlined in Scheme 1. To address the potential instability of the conjugated diene group, we propose synthesizing alternapyrone via a Stille coupling in the final steps. This strategy allows alternapyrone to be retrosynthetically traced back to alkenyl iodide (**2**) and alkenyl stannane (**3**). The α-pyrone core can be constructed through a retro Diels–Alder reaction, which simplifies its derivation from compound (**4**). This intermediate contains a methylene-protected group and can be converted into allyl iodide (**2**). Intermediate (**4**) can be synthesized via an aldol reaction between chiral aldehyde (**6**) and compound (**5**). In parallel, the long-chain stannane (**3**) can be readily obtained using a samarium diiodide-mediated Kagan–Molander coupling reaction between aldehyde (**7**) and allyl bromide (**8**). Following this retrosynthetic pathway, the complex structure of alternapyrone can be systematically constructed from simpler, commercially available compounds, ensuring precise stereochemical control at each step.

## 2. Results and Discussion

**Scheme 2:**
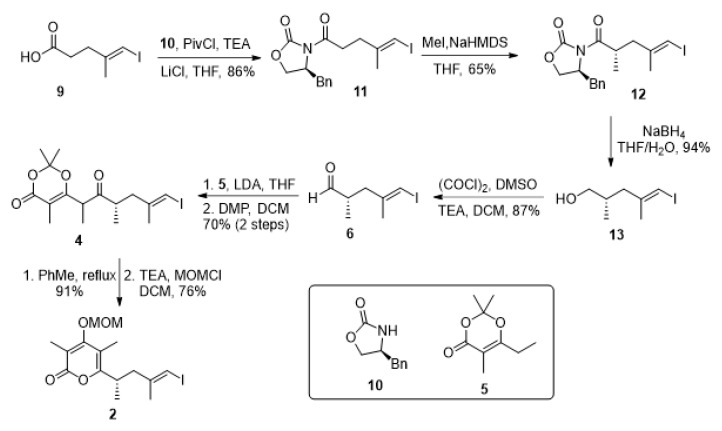
Synthesis of alkenyl iodide **2**.

Our synthesis, as shown in Scheme 2, is commenced with the preparation of vinyl iodide (**2**) starts from the known acid **9** [11]. Thus, the condensation of acid (**9**) with (*S*)-4-benzyl-2-azolidinone (**10**) produced the acyloxazolidinone **11** in 86% yield. Subsequent alkylation of the resulting acyloxazolidinone (**11**) using methyl iodide in the presence of NaHMDS proceeded with excellent diastereocontrol, providing the methylated adduct (**12**) as a single isomer in 65% yield [12]. The Evans auxiliary was then removed using sodium borohydride, and the resulting primary alcohol was oxidized under Swern oxidation conditions [13] to generate the corresponding aldehyde (**6**) in 82% yield over two steps. This aldehyde was then added to a lithio derivative of the cyclic compound (**5**), generated in situ from (**5**) and LDA at −78 °C to give the corresponding secondary alcohol intermediate [14] which was directly oxidized with the Dess−Martin periodinane reagent [15] to furnish ketone (**4**) in 70% yield over two steps. A Retro-Diels–Alder reaction of ketone (**4**) proceeded smoothly in refluxing toluene, providing the corresponding α-pyrone derivative in 91% yield [16], which was subsequently converted into fragment (**2**) using MOMCl (chloromethyl methyl ether) in the presence of triethylamine.

**Scheme 3:**
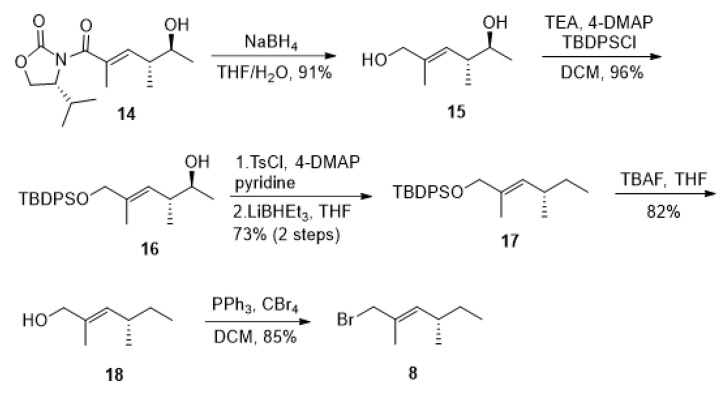
Synthesis of allyl bromide **8**.

The synthesis of allyl bromide (**8**) commenced with the enantiomerically pure alcohol (**14**), which was prepared via the Mukaiyama aldol reaction (Scheme 3) [17]. The auxiliary was reductively removed using sodium borohydride, providing diol (**15**) in 91% yield. The primary alcohol in diol (**15**) was selectively protected as a TBDPS-ether, giving compound (**16**). Subsequent tosylation of the secondary alcohol in (**16**), followed by deoxygenation using lithium triethylborohydride [18], afforded the deoxygenated product (**17**) in 73% yield over two steps. Removal of the TBDPS group with tetrabutylammonium fluoride (TBAF) in THF afforded the primary alcohol (**18**) in 82% yield. This primary alcohol (**18**) was then subjected to the Appel reaction with triphenylphosphine and carbon tetrabromide, furnishing allylic bromide (**8**) in 85% yield [19].

**Scheme 4:**
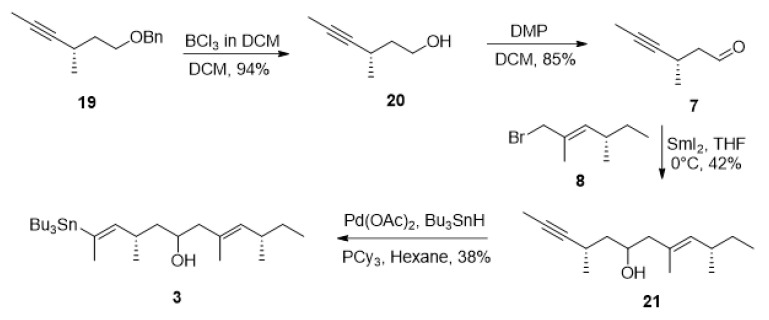
Synthesis of alkenyl tin **3**.

With allyl bromide (**8**) in hand, we turned our attention to the synthesis of fragment (**3**, Scheme 4). Deprotection of the benzyl group [20] from the known compound (**19**) [21] was achieved using boron trichloride. The exposed primary hydroxyl group was subsequently oxidized with Dess–Martin periodinane (DMP) to afford the corresponding aldehyde (**7**) [15]. A mixture of allyl bromide (**8**) and aldehyde (**7**) was then treated with samarium diiodide in THF, yielding the homoallylic alcohol (**21**) [22] as a mixture of two diastereomers (*d.r.* = 1.4:1). These diastereomers were separable by flash chromatography, giving a combined overall yield of 42% from aldehyde (**7**). Since the newly formed stereogenic center would ultimately be destroyed in a later stage of the synthesis, both isomers of (**21**) were deemed suitable for use in the total synthesis. To simplify the acquisition of clean spectra for the synthetic product, we chose to proceed with the major isomer in subsequent synthetic studies. Palladium-catalyzed hydrostannation of the alkynyl group in the major isomer of (**21**) afforded the alkenylstannane compound (**3**) with >95:5 *E*/*Z* selectivity [23].

**Scheme 5:**
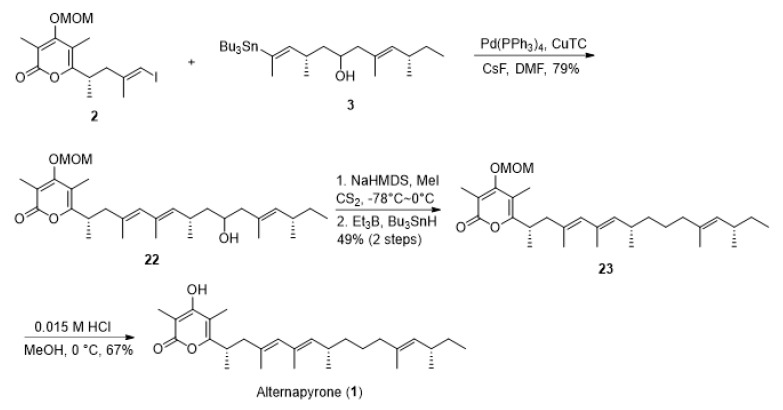
Completion of the synthesis of alternapyrone (**1**).

With both key intermediates (**2**) and (**3**) in hand, our focus shifted to the critical Stille cross-coupling reaction to join these two units, which is outlined in Scheme 5. The treatment of alkenylstannane (**3**) and alkenyl iodide (**2**) in the presence of tetrakis (triphenylphosphine)palladium, CuTC (copper thiophene-2-carboxylate), and cesium fluoride proceeded smoothly, yielding the desired dienyl adduct (**22**) [24]. The exposed hydroxyl group in (**22**) then underwent a deoxygenation process. First, reaction of (**22**) with iodomethane and carbon disulfide in the presence of NaHMDS formed a xanthate intermediate. This intermediate subsequently underwent a Barton–McCombie deoxygenation reaction using tributyltin hydride and triethylborane as the radical initiator, producing the corresponding hydrocarbon (**23**) [25]. Finally, the MOM protecting group in (**23**) was removed under mild acidic conditions [26], resulting in the formation of alternapyrone (**1**) with a yield of 67%. The structure of the synthetic alternapyrone (**1**) was confirmed through comprehensive spectroscopic analysis. The HRMS, ^1^H NMR, and ^13^C NMR data matched closely with reported research values for natural alternapyrone (see Supplementary Materials for details). The agreement in chemical shifts and coupling constants provided strong evidence for the successful synthesis. Additionally, further measurement of the CD spectrum of synthetic alternapyrone, along with a comparison to the calculated spectra [27,28] of the two possible enantiomers, confirmed the configuration of the synthetic sample. Consequently, the structure of alternapyrone (**1**) was unambiguously assigned as depicted in Figure 2b.

## 3. Materials and Methods

### 3.1. General

All reactions were conducted under an atmosphere of dry nitrogen or argon. Oxygen and/or moisture-sensitive reagents were transferred appropriately. All reaction solvents were purified and dried before use. Flash column chromatography was performed using the indicated solvents on silica gel 60 (230–400 mesh ASTM E. Qingdao, Tsingtao, China). The reactions were monitored using thin-layer chromatography. ^1^H- and ^13^C-NMR were taken in CDCl_3_ at 400 MHz and 75.0 MHz (Bruker, Karlsruhe, Germany). Chemical shifts were reported in parts per million (ppm) using the solvent resonance internal standard (deuterochloroform, 7.26 and 77.16 ppm). Data are reported as follows: chemical shift, multiplicity (ovrlp = overlapping, s = singlet, d = doublet, t = triplet, q = quartet, m = multiplet, br = broad), coupling constant, and integration. The ratios of diastereomers (dr) were determined by ^1^H-NMR (400 MHz) operating at a signal/noise ratio of >200:1. High-resolution mass spectra were measured on an ABI Q-star Elite (Beijing, China). Optical rotations were recorded on a Rudolph AutoPol-I polarimeter (Shanghai, China) at 589 nm with a 50 mm cell. Data are reported as follows: specific rotation (c (g/100 mL), solvent).

### 3.2. General Experimental Procedures

(*S,E*)-4-benzyl-3-(5-iodo-4-methylpent-4-enoyl)oxazolidin-2-one (**11**)

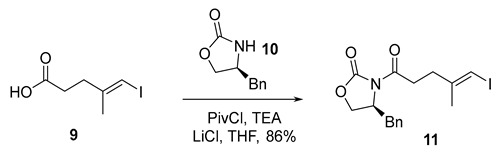



To a cooled (−10 °C) solution of compound **9** (3.68 g, 15.34 mmol, 1.0 equiv.) in anhydrous THF (80 mL, 0.19 M) under argon, TEA (5.3 mL, 38.35 mmol, 2.5 equiv.) and PivCl (1.9 mL, 15.34 mmol, 1.0 equiv.) were added. After being stirred at −10 °C for 1 h, LiCl (976 mg, 23.01 mmol, 1.5 equiv.) and (*S*)-4-benzyl-2-azolidinone **10** (2.66 g, 15.03 mmol, 0.98 equiv.) were added. The reaction mixture was warm to ambient temperature, at which point the starting material was consumed, as verified by TLC analysis, and the aqueous saturated NH_4_Cl (20 mL) was slowly added at 0 °C. The organic layer was separated and the aqueous phase was extracted with EtOAc (3 × 50 mL). The combined organic phases were washed with brine (20 mL) and dried over anhydrous Na_2_SO_4_. The solvent was removed in vacuo and the crude product was purified by flash chromatography on silica gel (20% EtOAc/hexanes) to give compound **11** as a colorless oil (5.26 g, 86%).

**R_f_** = 0.40 (20% EtOAc/hexanes), UV and PMA stain.
${\left[\alpha \right]}_{D}^{20}$ = +25.5 (*c* 0.50, CHCl_3_);
**HRMS** (ESI, *m*/*z*) for C_16_H_18_INO_3_Na^+^ [M + Na]^+^: calcd. 422.0224; found: 422.0225.
**^1^H NMR** (400 MHz, CDCl_3_) δ 7.37–7.24 (m, 3H), 7.23–7.17 (m, 2H), 6.03 (q, *J* = 1.1 Hz, 1H), 4.67 (ddt, *J* = 12.8, 7.1, 3.3 Hz, 1H), 4.26–4.14 (m, 2H), 3.28 (dd, *J* = 13.4, 3.4 Hz, 1H), 3.22–2.98 (m, 2H), 2.77 (dd, *J* = 13.4, 9.6 Hz, 1H), 2.68–2.52 (m, 2H), 1.90 (d, *J* = 1.0 Hz, 3H).
**^13^C NMR** (101 MHz, CDCl_3_) δ 172.1, 153.5, 146.2, 135.3, 129.5, 129.1, 127.5, 76.4, 66.4, 55.3, 38.0, 33.9, 33.8, 24.1.

(*S*)-4-benzyl-3-((*S,E)*-5-iodo-2,4-dimethylpent-4-enoyl)oxazolidin-2-one (**12**)

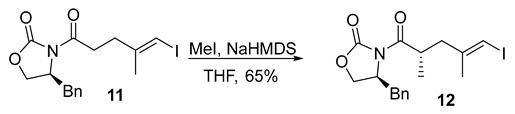



To a stirred solution of **11** (3.30 g, 8.27 mmol, 1.0 equiv.) in anhydrous THF (50 mL), NaHMDS (5.4 mL, 10.75 mmol, 2.0 M in THF. 1.3 equiv.) was added dropwise at −78 °C under argon. After being stirred for 30 min, MeI (0.77 mL, 12.41 mmol, 1.5 equiv.) was added. This mixture was cooled to –78 °C, and stirred for 1 h, then slowly warmed to −40 °C and stirred at this temperature for 3 h, at which point **11** had been consumed,, as verified by TLC analysis; then, aqueous saturated NH_4_Cl (20 mL) was slowly added. The aqueous phase was extracted with MTBE (3 × 50 mL). The combined organic phases were washed with H_2_O (10 mL) and brine (10 mL) in sequence, and then dried over anhydrous Na_2_SO_4_. The solvent was removed in vacuo and the crude product was purified by flash chromatography on silica gel (12% EtOAc/hexanes) to give compound **11** as a colorless oil (2.22 g, 65%).

**R_f_** = 0.40 (20% EtOAc/hexanes), UV and PMA stain.
${\left[\alpha \right]}_{D}^{20}$ = +35.3 (*c* 0.50, CHCl_3_);
**HRMS** (ESI, *m/z*) for C_17_H_20_INO_3_Na^+^ [M + Na]^+^: calcd. 436.0380; found: 436.0380.
**^1^H NMR** (500 MHz, CDCl_3_) δ 7.36–7.25 (m, 3H), 7.23–7.18 (m, 2H), 5.97 (s, 1H), 4.64 (tt, *J* = 10.5, 3.1 Hz, 1H), 4.26–4.14 (m, 2H), 4.05–3.95 (m, 1H), 3.25 (dd, *J* = 13.4, 3.3 Hz, 1H), 2.77 (dd, *J* = 13.4, 9.6 Hz, 1H), 2.66 (dd, *J* = 13.5, 7.2 Hz, 1H), 2.29 (dd, *J* = 13.6, 7.4 Hz, 1H), 1.86 (s, 3H), 1.19 (d, *J* = 6.8 Hz, 3H).
**^13^C NMR** (101 MHz, CDCl_3_) δ 176.2, 153.2, 145.3, 135.3, 129.6, 129.1, 127.5, 77.3, 66.3, 55.5, 43.2, 38.0, 36.0, 23.9, 17.1.

(*S*,*E*)-5-iodo-2,4-dimethylpent-4-en-1-ol (**13**)

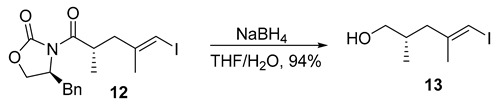



To a stirred solution of compound **12** (1.11 g, 2.69 mmol, 1.0 equiv.) in THF (20 mL)/H_2_O (10 mL) at 0 °C, NaBH_4_ (508 mg, 13.44 mmol, 5.0 equiv.) was added. The reaction mixture was allowed to warm to ambient temperature and stirred continuously for 3 h, at which point **12** had been consumed, as verified by TLC analysis; the reaction was quenched by adding aqueous saturated NH_4_Cl (5 mL) slowly at 0 °C. The organic layer was separated and the aqueous phase was extracted with MTBE (3 × 20 mL). The combined organic phases were washed with brine (10 mL) and dried over anhydrous Na_2_SO_4_. The solvent was removed in vacuo and the crude product was purified by flash chromatography on silica gel (25% EtOAc/hexanes) to give compound **13** as a colorless oil (607 mg, 94%). Spectroscopic data were in agreement with those reported previously [29].

**R_f_** = 0.40 (25% EtOAc/hexanes), UV and PMA stain.
${\left[\alpha \right]}_{D}^{28}$ = −9.6 (*c* 1.25, CHCl_3_);
**^1^H NMR** (500 MHz, CDCl_3_) δ 5.92–5.86 (m, 1H), 3.52–3.38 (m, 2H), 2.35 (ddd, *J* = 13.5, 6.1, 1.2 Hz, 1H), 2.01 (ddd, *J* = 13.5, 8.4, 0.9 Hz, 1H), 1.92–1.81 (m, 1H), 1.83 (d, *J* = 1.1 Hz, 3H), 0.87 (d, *J* = 6.7 Hz, 3H).
**^13^C NMR** (101 MHz, CDCl_3_) δ 146.7, 75.8, 67.8, 43.7, 33.9, 23.9, 16.4.

(*S*,*E*)-5-iodo-2,4-dimethylpent-4-enal (**6**)

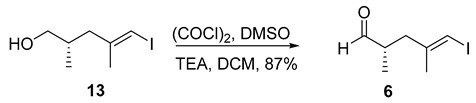



To a solution of (COCl)_2_ (0.18 mL, 2.13 mmol, 5.0 equiv.) in anhydrous DCM (12 mL) at −78 °C, DMSO (0.30 mL, 4.25 mmol, 10.0 equiv.) was added dropwise. After being stirred for 10 min, a solution of compound **13** (102 mg, 0.43 mmol, 1.0 equiv.) in DCM (1 mL) was added, and the mixture was stirred at −78 °C for 20 min. The mixture was warmed to −40 °C and kept for 20 min, before it was re-cooled to −78 °C, and then TEA (1.1 mL, 7.65 mmol, 18.0 equiv.) was added dropwise. The reaction mixture was allowed to warm to ambient temperature and stirred continuously for 2 h, at which point **13** had been consumed, as verified by TLC analysis; then, the reaction was quenched by slowly adding aqueous saturated NH_4_Cl (3 mL) at 0 °C. The organic layer was separated and the aqueous phase was extracted with EA (3 × 10 mL). The combined organic phases were washed with brine (5 mL) and dried over anhydrous Na_2_SO_4_. The solvent was removed in vacuo and the crude product was purified by flash chromatography on silica gel (20% EtOAc/hexanes) to give compound **8** as a colorless oil (88 mg, 87%). Spectroscopic data were in agreement with those reported previously [30].

**R_f_** = 0.50 (20% EtOAc/hexanes), UV and PMA stain.
${\left[\alpha \right]}_{D}^{21}$ = −22.7 (*c* 0.72, CHCl_3_);
**^1^H NMR** (500 MHz, CDCl_3_) δ 9.62 (d, *J* = 1.5 Hz, 1H), 5.99–5.95 (m, 1H), 2.64 (ddd, *J* = 13.9, 6.0, 1.3 Hz, 1H), 2.59–2.48 (m, 1H), 2.19 (ddd, *J* = 13.9, 8.4, 0.8 Hz, 1H), 1.82 (d, *J* = 1.1 Hz, 3H), 1.06 (d, *J* = 7.0 Hz, 3H).
**^13^C NMR** (126 MHz, CDCl_3_) δ 203.8, 144.6, 77.2, 44.3, 40.2, 23.8, 13.2.

6-((4*S*,*E*)-7-iodo-4,6-dimethyl-3-oxohept-6-en-2-yl)-2,2,5-trimethyl-4H-1,3-dioxin-4-one (**4**)

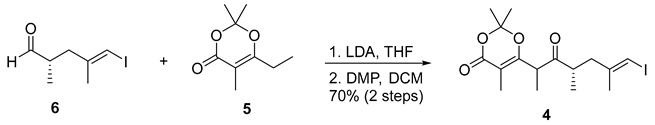



To a cooled (−78 °C) stirred solution of DIPA (0.33 mL, 2.35 mmol, 7.0 equiv.) in anhydrous THF (7 mL), *n*BuLi (1.5 mL, 2.35 mmol, 1.6 M in hexane, 7.0 equiv.) was added dropwise. After being stirred at −78 °C for 15 min, a solution of compound **5** (400 mg, 2.35 mmol, 7.0 equiv.) in THF (2 mL) was added dropwise and the mixture was kept at −78 °C for 30 min before a solution of compound **6** (80 mg, 0.336 mmol, 1.0 equiv.) in THF (1 mL) was added dropwise. The mixture was stirred at −78 °C overnight, at which point all the starting materials had been consumed, as verified by TLC analysis; then, the reaction was quenched by slowly adding aqueous saturated NH_4_Cl (3 mL). After acidified to pH 1~2 with 1 M HCl, the organic layer was separated and the aqueous phase was extracted with EA (3 × 50 mL). The combined organic phases were washed with brine (10 mL) and dried over anhydrous Na_2_SO_4_. The solvent was removed in vacuo and the crude product was purified by flash chromatography on silica gel (33% EtOAc/hexanes), which was directly used in the next step.

To a cooled (0 °C) solution of the above intermediate compound (113 mg, 0.277 mmol, 1.0 equiv.) in DCM (6 mL, 0.05 M.), DMP (590 mg, 1.39 mmol, 5.0 equiv.) was added. The mixture was allowed to slowly warm to ambient temperature for 1 h, at which point the starting material had been consumed, as verified by TLC analysis; then, the reaction was quenched by slowly adding aqueous saturated Na_2_S_2_O_3_ (10 mL) and NaHCO_3_ (10 mL) at 0 °C. The organic layer was separated and the aqueous phase was extracted with DCM (3 ×50 mL). The combined organic phases were washed with brine (10 mL) and dried over anhydrous Na_2_SO_4_. The solvent was removed in vacuo and the crude product was purified by flash chromatography on silica gel (20% EtOAc/hexanes) to give compound **4** as a colorless oil (95 mg, *dr* =1:1.5, 70% for two steps).

**R_f_** = 0.40 (20% EtOAc/hexanes), UV and PMA stain.
**HRMS** (ESI, *m/z*) for C_16_H_23_IO_4_Na^+^ [M + Na]^+^: calcd. 429.0533; found: 429.0521.
**^1^H NMR** (400 MHz, CDCl_3_) δ 5.95–5.90 (m, 2.5H), 3.76 (q, *J* = 6.9 Hz, 1H), 3.54 (q, *J* = 6.9 Hz, 1.5H), 2.94–2.78 (m, 2.5H), 2.56 (ddd, *J* = 14.0, 6.4, 1.1 Hz, 1H), 2.47 (ddd, *J* = 13.4, 7.8, 1.0 Hz, 1.5H), 2.23–2.13 (m, 2.5H), 1.91 (s, 3H), 1.90 (s, 4.5H), 1.82 (d, *J* = 1.1 Hz, 4.5H), 1.76 (d, *J* = 1.1 Hz, 3H), 1.64 (s, 3H), 1.63 (s, 4.5H), 1.63–1.59 (m, 7.5H), 1.26 (d, *J* = 6.9 Hz, 3H), 1.23 (d, *J* = 6.9 Hz, 4.5H), 1.05 (d, *J* = 7.1 Hz, 3H), 1.02 (d, *J* = 6.7 Hz, 4.5H).
**^13^C NMR** (101 MHz, CDCl_3_) δ 209.1, 208.2, 162.7, 162.6, 162.4, 144.7, 144.7, 105.6, 105.5, 102.5, 102.3, 77.9, 77.6, 48.8, 46.8, 44.2, 42.8, 42.3, 41.9, 26.3, 26.0, 24.0, 24.0, 23.9, 23.8, 17.4, 16.7, 12.4, 12.3, 10.4, 10.4.

(*S*,*E*)-6-(5-iodo-4-methylpent-4-en-2-yl)-4-(methoxymethoxy)-3,5-dimethyl-2H-pyran-2-one (**2**)

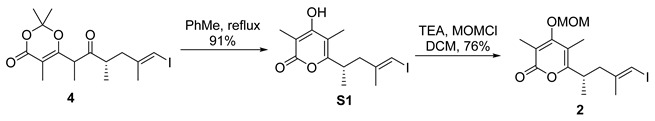



To a flask equipped with a reflux condenser, toluene (54 mL) was added, and heated to 110 °C, to which a solution of compound **4** (23 mg, 0.0566 mmol, 1.0 equiv.) in toluene (2 mL) was added dropwise. The mixture was then refluxed for 65 h and then cooled to ambient temperature. The solvent was removed in vacuo and the crude product was purified by flash chromatography on silica gel (100% EtOAc/hexanes) to give the intermediate compound **S1** as a white solid (18 mg, 91%).

**R_f_** = 0.50 (100% EtOAc/hexanes), UV and PMA stain.
${\left[\alpha \right]}_{D}^{20}$ = +88.1 (*c* 0.50, CHCl_3_);
**HRMS** (ESI, *m/z*) for C_13_H_17_IO_3_Na^+^ [M + Na]^+^: calcd. 371.0115; found: 371.0109.
**^1^H NMR** (400 MHz, CDCl_3_) δ 5.90 (q, *J* = 1.1 Hz, 1H), 3.16–3.02 (m, 1H), 2.57 (ddd, *J* = 13.7, 8.4, 1.0 Hz, 1H), 2.37 (ddd, *J* = 13.7, 6.7, 1.0 Hz, 1H), 2.01 (s, 3H), 1.96 (s, 3H), 1.79 (d, *J* = 1.1 Hz, 3H), 1.17 (d, *J* = 6.9 Hz, 3H).
**^13^C NMR** (101 MHz, CDCl_3_) δ 166.3, 165.0, 160.5, 145.1, 106.9, 98.5, 77.5, 43.9, 33.2, 24.1, 18.1, 9.7, 8.8.

To a cooled (0 °C) solution of intermediate compound **S1** (128 mg, 0.368 mmol, 1.0 equiv.) in anhydrous DCM (30 mL, 0.012 M), TEA (0.26 mL, 1.84 mmol, 5.0 equiv.) was added dropwise. After being stirred at 0 °C for 10 min, MOMCl (0.14 mL, 1.84 mmol, 5.0 equiv.) was added dropwise. The reaction mixture was allowed to warm to ambient temperature and vigorous stirring was continued for 12 h, at which point TLC analysis indicated that only a trace of aldehyde **S1** remained. The reaction was quenched by slowly adding H_2_O at 0 °C. The organic layer was separated and the aqueous phase was extracted with DCM (3 × 30 mL). The combined organic phases were washed with brine (10 mL) and dried over anhydrous Na_2_SO_4_. The solvent was removed in vacuo and the crude product was purified by flash chromatography on silica gel (33% EtOAc/hexanes) to give compound **2** as a white solid (110 mg, 76%).

**R_f_** = 0.40 (33% EtOAc/hexanes), UV and PMA stain.
${\left[\alpha \right]}_{D}^{20}$ = +109.1 (*c* 0.50, CHCl_3_);
**HRMS** (ESI, *m/z*) for C_15_H_21_IO_4_Na^+^ [M + Na]^+^: calcd. 415.0377; found: 415.0370.
**^1^H NMR** (500 MHz, CDCl_3_) δ 5.88 (q, *J* = 1.1 Hz, 1H), 5.04 (s, 2H), 3.56 (s, 3H), 3.11–3.00 (m, 1H), 2.58 (ddd, *J* = 13.7, 8.3, 1.0 Hz, 1H), 2.39 (ddd, *J* = 13.7, 6.7, 1.0 Hz, 1H), 2.01 (s, 3H), 1.92 (s, 3H), 1.80 (d, *J* = 1.1 Hz, 3H), 1.18 (d, *J* = 6.8 Hz, 3H).
**^13^C NMR** (101 MHz, CDCl_3_) δ 166.3, 165.7, 160.2, 145.2, 110.1, 109.1, 99.2, 77.4, 58.0, 44.0, 33.5, 24.1, 17.9, 11.0, 10.5.

(4*R*,5*S*,*E*)-2,4-dimethylhex-2-ene-1,5-diol (**15**)

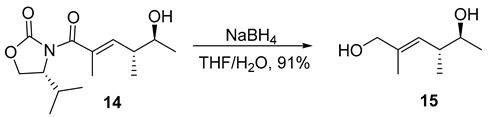



To a cooled (0 °C) stirred solution of compound **14** (4.30 g, 15.98 mmol, 1.0 equiv.) in THF (80 mL)/H_2_O (40 mL), NaBH_4_ (2.42 g, 63.92 mmol, 4.0 equiv.) was added. The reaction mixture was allowed to warm to ambient temperature and stirred continuously for 2 h, at which point the starting material had been consumed, as verified by TLC analysis; then, the reaction was quenched by adding aqueous saturated NH_4_Cl (20 mL) slowly at 0 °C. The organic layer was separated and the aqueous phase was extracted with MTBE (3 × 80 mL). The combined organic phases were washed with brine (20 mL) and dried over anhydrous Na_2_SO_4_. The solvent was removed in vacuo and the crude product was purified by flash chromatography on silica gel (150% EtOAc/hexanes) to give compound **15** as a colorless oil (2.10 g, 91%).

**R_f_** = 0.50 (150% EtOAc/hexanes), UV and PMA stain.
${\left[\alpha \right]}_{D}^{20}$ = +20.0 (*c* 0.50, CHCl_3_);
**HRMS** (ESI, *m/z*) for C_8_H_16_O_2_Na^+^ [M + Na]^+^: calcd. 167.1043; found: 167.1043.
**^1^H NMR** (400 MHz, CDCl_3_) δ 5.32–5.23 (m, 1H), 4.02 (d, *J* = 1.5 Hz, 2H), 3.59–3.48 (m, 1H), 2.45–2.31 (m, 1H), 2.03–1.82 (m, 2H), 1.69 (d, *J* = 1.5 Hz, 3H), 1.18 (d, *J* = 6.2 Hz, 3H), 0.95 (d, *J* = 6.8 Hz, 3H).
**^13^C NMR** (101 MHz, CDCl_3_) δ 137.1, 127.9, 72.0, 68.6, 40.3, 20.5, 17.1, 14.3.

(2*S*,3*R*,*E*)-6-((*tert*-butyldiphenylsilyl)oxy)-3,5-dimethylhex-4-en-2-ol (**16**)

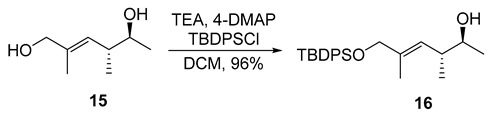



To a cooled (0 °C) stirred solution of compound **15** (2.14 g, 14.87 mmol, 1.0 equiv.) in DCM (100 mL), 4-DMAP (363 mg, 2.97 mmol, 0.2 equiv.), TEA (3.1 mL, 22.31 mmol, 1.5 equiv.) and TBDPSCl (4.6 mL, 17.84 mmol, 1.2 equiv.).were sequentially added. The reaction mixture was allowed to warm to ambient temperature and stirred continuously for 2 h, at which point the starting material had been consumed, as verified by TLC analysis; then, the reaction was quenched by adding H_2_O (20 mL) slowly at 0 °C. The organic layer was separated and the aqueous phase was extracted with EA (3 × 40 mL). The combined organic phases were washed with brine (20 mL) and dried over anhydrous Na_2_SO_4_. The solvent was removed in vacuo and the crude product was purified by flash chromatography on silica gel (12.5% EtOAc/hexanes) to give compound **16** as a colorless oil (5.46 g, 96%). Spectroscopic data were in agreement with those reported previously [31].

**R_f_** = 0.50 (12.5% EtOAc/hexanes), UV and PMA stain.
${\left[\alpha \right]}_{D}^{22}$ = +26.0 (*c* 1.00, CHCl_3_);
**^1^H NMR** (400 MHz, CDCl_3_) δ 7.71–7.63 (m, 4H), 7.46–7.33 (m, 6H), 5.27 (dq, *J* = 10.0, 1.5 Hz, 1H), 4.08 (d, *J* = 1.5 Hz, 2H), 3.53–3.42 (m, 1H), 2.45–2.31 (m, 1H), 1.64 (d, *J* = 1.4 Hz, 3H), 1.17 (d, *J* = 6.2 Hz, 3H), 1.06 (s, 9H), 0.94 (d, *J* = 6.8 Hz, 3H).
**^13^C NMR** (101 MHz, CDCl_3_) δ 137.0, 135.7, 133.9, 129.8, 127.8, 126.5, 71.9, 68.9, 40.3, 27.0, 20.1, 19.4, 17.0, 14.2.

(*S*,*E*)-*tert*-butyl((2,4-dimethylhex-2-en-1-yl)oxy)diphenylsilane (**17**)

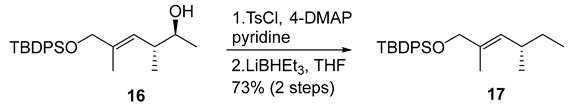



To a cooled (0 °C) stirred solution of compound **16** (5.34 g, 13.97 mmol, 1.0 equiv.) in pyridine (140 mL, 0.1 M), 4-DMAP (171 mg, 1.4 mmol, 0.10 equiv.) and TsCl (13.32 g, 69.85 mmol, 5.0 equiv.) were added. The reaction mixture was allowed to warm to ambient temperature and stirred for 6 h, at which point the starting material had been consumed, as verified by TLC analysis; then, the reaction was quenched by adding aqueous saturated NH_4_Cl (40 mL) slowly at 0 °C. The organic layer was separated and the aqueous phase was extracted with DCM (3 × 80 mL). The combined organic phases were washed with aqueous saturated NaHCO_3_ (2 × 40 mL) and brine (40 mL), and dried over anhydrous Na_2_SO_4_. The solvent was removed in vacuo and the crude product was directly used in the next step without further purification.

To a cooled (0 °C) stirred solution of the above intermediate compound in anhydrous THF (140 mL, 0.1M), LiBHEt_3_ (84 mL, 83.82 mmol, 6.0 equiv.) was added dropwise. The reaction mixture was allowed to warm to ambient temperature and stirred continuously for 10 h, at which point the starting material had been consumed, as verified by TLC analysis; then, the reaction was quenched by adding aqueous saturated NH_4_Cl (20 mL) slowly at 0 °C. The organic layer was separated and the aqueous phase was extracted with EA (3 × 40 mL). The combined organic phases were washed with brine (10 mL), and then dried over anhydrous Na_2_SO_4_. The solvent was removed in vacuo and the crude product was purified by flash chromatography on silica gel (100% hexanes) to give compound **17** as a colorless oil (3.73 g, 73% for two steps).

**R_f_** = 0.80 (10% EtOAc/hexanes), UV and PMA stain.
${\left[\alpha \right]}_{D}^{20}$ = +15.0 (*c* 0.10, CHCl_3_);
**HRMS** (ESI, *m/z*) for C_24_H_34_OSiNa^+^ [M + Na]^+^: calcd. 389.2271; found: 389.2273
**^1^H NMR** (500 MHz, CDCl_3_) δ 7.74–7.66 (m, 4H), 7.45–7.33 (m, 6H), 5.22–5.15 (m, 1H), 4.06 (d, *J* = 1.5 Hz, 2H), 2.36–2.24 (m, 1H), 1.62 (d, *J* = 1.4 Hz, 3H), 1.39–1.17 (m, 2H), 1.06 (s, 9H), 0.93 (d, *J* = 6.6 Hz, 3H), 0.85 (t, *J* = 7.4 Hz, 3H).
**^13^C NMR** (101 MHz, CDCl_3_) δ 135.7, 134.2, 132.8, 131.3, 129.6, 127.7, 69.4, 33.7, 30.5, 27.0, 20.9, 19.5, 13.9, 12.1.

(*S*,*E*)-2,4-dimethylhex-2-en-1-ol (**18**)

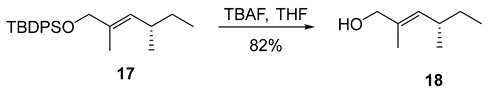



To a cooled (0 °C) stirred solution of compound **17** (304 mg, 0.83 mmol, 1.0 equiv.) in THF (3 mL), TBAF (1.2 mL, 1.23 mmol, 1 M in THF, 1.5 equiv.) was added. The reaction mixture was allowed to warm to ambient temperature and stirred continuously for 2 h, at which point the starting material had been consumed, as verified by TLC analysis; then, the reaction was quenched by adding H_2_O (1 mL) slowly at 0 °C. The organic layer was separated and the aqueous phase was extracted with Et_2_O (3 × 5 mL). The combined organic phases were washed with brine (2 mL), and then dried over anhydrous Na_2_SO_4_. The solvent was removed in vacuo at 0 °C and the crude product was purified by flash chromatography on silica gel (20% Et_2_O/pentane) to give compound **18** as a colorless oil (87 mg, 82%). Spectroscopic data were in agreement with those reported previously [14].

**R_f_** = 0.40 (20% Et_2_O/pentane), PMA stain.
${\left[\alpha \right]}_{D}^{25}$ = +34.3 (*c* 1.05, CHCl_3_);
**^1^H NMR** (500 MHz, CDCl_3_) δ 5.17 (dq, *J* = 9.5, 1.3 Hz, 1H), 4.00 (d, *J* = 1.2 Hz, 2H), 2.36–2.21 (m, 1H), 1.67 (d, *J* = 1.4 Hz, 3H), 1.41—1.15 (m, 2H), 0.93 (d, *J* = 6.7 Hz, 3H), 0.83 (t, *J* = 7.4 Hz, 3H).
**^13^C NMR** (101 MHz, CDCl_3_) δ 133.5, 132.9, 69.3, 33.9, 30.4, 20.8, 14.0, 12.1.

(*S*,*E*)-1-bromo-2,4-dimethylhex-2-ene (**8**)

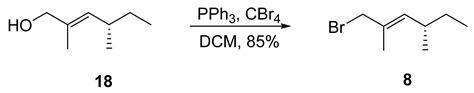



To a cooled (0 °C) stirred solution of compound **18** (434 mg, 3.39 mmol, 1.0 equiv.) in anhydrous DCM (26 mL, 0.13 M), CBr_4_ (1.46 g, 4.41 mmol, 1.3 equiv.) and PPh_3_ (1.33 g, 5.09 mmol, 1.5 equiv.) were added. The reaction mixture was allowed to warm to ambient temperature and stirred for 0.5 h. The solvent was removed in vacuo at 0 °C and the crude product was purified by flash chromatography on silica gel (pentane) to give compound **8** as a colorless oil (548 mg, 85%).

**R_f_** = 0.90 (100% pentane), UV and PMA stain.
${\left[\alpha \right]}_{D}^{20}$ = +9.7 (*c* 0.50, CHCl_3_);
**^1^H NMR** (400 MHz, CDCl_3_) δ 5.39–5.32 (m, 1H), 3.99–3.95 (m, 2H), 2.32–2.17 (m, 1H), 1.76 (d, *J* = 1.4 Hz, 3H), 1.43–1.15 (m, 2H), 0.93 (d, *J* = 6.6 Hz, 3H), 0.83 (t, *J* = 7.4 Hz, 3H).
**^13^C NMR** (101 MHz, CDCl_3_) δ 137.9, 130.9, 42.3, 34.6, 30.2, 20.3, 15.0, 12.0.

(*S*)-3-methylhex-4-yn-1-ol (**20**)

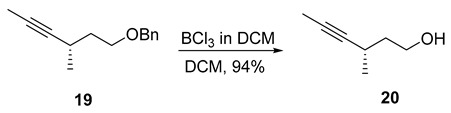



To a cooled (−78 °C) stirred solution of compound **19** (1.53 g, 7.57 mmol, 1.0 equiv.) in anhydrous DCM (76 mL, 0.1 M), BCl_3_ (23 mL, 22.71 mmol, 1.0 M in DCM. 3.0 equiv.) was added dropwise. The reaction mixture was continuously stirred at −78 °C for 1 h before it was quenched by adding MeOH (20 mL) and aqueous saturated NaHCO_3_ (20 mL) slowly. The organic layer was separated and the aqueous phase was extracted with Et_2_O (3 × 50 mL). The combined organic phases were washed with brine (10 mL), and then dried over anhydrous Na_2_SO_4_. The solvent was removed in vacuo at 0 °C and the crude product was purified by flash chromatography on silica gel (33.3% Et_2_O/pentane) to give compound **20** as a colorless oil (798 mg, 94%).

**R_f_** = 0.30 (33.3% Et_2_O/pentane), PMA stain.
${\left[\alpha \right]}_{D}^{20}$ = +21.7 (*c* 0.50, CHCl_3_);
**HRMS** (ESI, *m/z*) for C_7_H_12_ONa^+^ [M + Na]^+^: calcd. 135.0780; found: 135.0783.
**^1^H NMR** (500 MHz, CDCl_3_) δ 3.83–3.71 (m, 2H), 2.61–2.48 (m, 1H), 1.85 (s, 1H), 1.77 (d, *J* = 2.4 Hz, 3H), 1.72–1.54 (m, 2H), 1.15 (dd, *J* = 6.9, 3H).
**^13^C NMR** (101 MHz, CDCl_3_) δ 83.3, 76.7, 61.5, 39.8, 23.1, 21.7, 3.6.

(S)-3-methylhex-4-ynal (**7**)

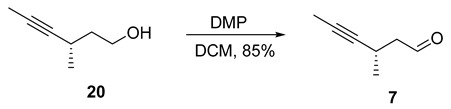



To a cooled (0 °C) stirred solution of compound **20** (300 mg, 2.68 mmol, 1.0 equiv.) dissolved in anhydrous DCM (27 mL, 0.1 M.), DMP (2.27 g, 5.36 mmol, 2.0 equiv.) was added. The reaction mixture was allowed to warm to ambient temperature and stirred continuously for 3 h, at which point the starting material had been consumed, as verified by TLC analysis; then, pentane (27 mL) was added and the reaction mixture was re-cooled to −50 °C. The mixture was rapidly filtered through a short pad of silica gel (25% Et_2_O/pentane, −50 °C). The combined organic phases were washed with aqueous saturated Na_2_S_2_O_3_ (2×30 mL), aqueous saturated NaHCO_3_ (2 × 30 mL) and brine (10 mL) in sequence, and then dried over anhydrous Na_2_SO_4_. The solvent was removed in vacuo at 0 °C and the crude product was purified by flash chromatography on silica gel (25% Et_2_O/pentane) to give compound **7** as a colorless oil (251 mg, 85%).

**R_f_** = 0.50 (25% Et_2_O/pentane), PMA stain.
${\left[\alpha \right]}_{D}^{20}$ = +12.0 (*c* 0.50, CHCl_3_);
**HRMS** (ESI, *m/z*) for C_7_H_10_ONa^+^ [M + Na]^+^: calcd. 133.0624; found: 133.0624.
**^1^H NMR** (500 MHz, CDCl_3_) δ 9.79 (t, *J* = 2.1 Hz, 1H), 2.98—2.86 (m, 1H), 2.53 (ddd, *J* = 16.5, 7.6, 2.2 Hz, 1H), 2.45 (ddd, *J* = 16.5, 6.3, 2.0 Hz, 1H), 1.77 (d, *J* = 2.4 Hz, 3H), 1.20 (d, *J* = 6.9 Hz, 3H).
**^13^C NMR** (101 MHz, CDCl_3_) δ 201.8, 81.7, 77.2, 50.4, 21.4, 20.9, 3.5.

(4*S*,10*S*,*E*)-4,8,10-trimethyldodec-8-en-2-yn-6-ol (**21**)

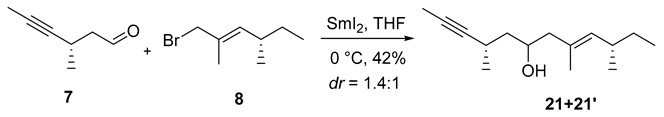



To a cooled (0 °C) stirred solution of compound **7** (560 mg, 5.08 mmol, 1.0 equiv.) and compound **8** (966 mg, 5.08 mmol, 1.0 equiv.) in THF (50 mL, 0.1 M) under argon, SmI_2_ (152 mL, 15.24 mmol, 0.1 M in THF, 3.0 equiv.) was added dropwise. After being stirred at 0 °C for 0.5 h, additional SmI_2_ (102 mL, 10.16 mmol, 0.1 M in THF, 2.0 equiv.) was added into the reaction system dropwise, and the resulting mixture was stirred at 0 °C for 12 h. The reaction was quenched by adding aqueous saturated NH_4_Cl (60 mL) slowly. The organic layer was separated and the aqueous phase was extracted with EA (3 × 100 mL). The combined organic phases were washed with aqueous saturated Na_2_S_2_O_3_ (80 mL) and brine (40 mL) in sequence, and then dried over anhydrous Na_2_SO_4_. The solvent was removed in vacuo and the crude product was purified by flash chromatography on silica gel (8.3% EtOAc/hexanes) to give compounds **21** and **21′** as colorless oils (474 mg, dr = 1.4:1, 42%).

**21: R_f_** = 0.40 (10% EtOAc/hexanes), PMA stain.
${\left[\alpha \right]}_{D}^{20}$ = +45.3 (*c* 0.10, CHCl_3_);
**HRMS** (ESI, *m/z*) for C_15_H_26_ONa^+^ [M+Na]^+^: calcd. 245.1876; found: 245.1876.
**^1^H NMR **(500 MHz, CDCl_3_) δ 5.01 (dq, *J* = 9.5, 1.3 Hz, 1H), 3.94 (tt, *J* = 8.6, 4.1 Hz, 1H), 2.76–2.65 (m, 1H), 2.33–2.23 (m, 1H), 2.18 (ddd, *J* = 13.2, 4.4, 1.1 Hz, 1H), 2.06 (ddd, *J* = 13.2, 9.0, 0.9 Hz, 1H), 1.91 (s, 1H), 1.80 (d, *J* = 2.4 Hz, 3H), 1.66 (d, *J* = 1.4 Hz, 3H), 1.51–1.39 (m, 2H), 1.40–1.28 (m, 1H), 1.27–1.18 (m, 1H), 1.16 (d, *J* = 7.0 Hz, 3H), 0.93 (d, *J* = 6.7 Hz, 3H), 0.84 (t, *J* = 7.4 Hz, 3H).
**^13^C NMR** (101 MHz, CDCl_3_) δ 135.7, 130.6, 83.4, 76.4, 66.8, 48.5, 44.4, 34.3, 30.5, 23.0, 22.1, 21.3, 16.5, 12.1, 3.7.
**21′: R_f_** = 0.50 (10% EtOAc/hexanes), PMA stain.
${\left[\alpha \right]}_{D}^{20}$ = +42.7 (*c* 0.10, CHCl_3_);
**HRMS** (ESI, *m/z*) for C_15_H_26_ONa^+^ [M+Na]^+^: calcd. 245.1876; found: 245.1876.
**^1^H NMR** (500 MHz, CDCl_3_) δ5.00 (dq, *J* = 9.5, 1.3 Hz, 1H), 3.84 (ddt, *J* = 8.9, 8.0, 4.4 Hz, 1H), 2.60–2.49 (m, 1H), 2.35–2.22 (m, 1H), 2.19 (ddd, *J* = 13.4, 4.1, 1.2 Hz, 1H), 2.02 (ddd, *J* = 13.3, 8.8, 0.9 Hz, 1H), 1.79 (d, *J* = 2.4 Hz, 3H), 1.65 (d, *J* = 1.4 Hz, 3H), 1.68–1.58 (m, 1H), 1.49 (ddd, *J* = 13.6, 6.5, 4.7 Hz, 1H), 1.39–1.28 (m, 1H), 1.26–1.15 (m, 1H), 1.17 (d, *J* = 6.9 Hz, 3H), 0.93 (d, *J* = 6.7 Hz, 3H), 0.84 (t, *J* = 7.4 Hz, 3H).
**^13^C NMR** (101 MHz, CDCl_3_) δ 135.5, 130.6, 83.8, 76.6, 67.5, 48.0, 44.0, 34.4, 30.5, 23.3, 21.6, 20.9, 16.5, 12.3, 3.6.

(2*E*,4*S*,8*E*,10*S*)-4,8,10-trimethyl-2-(tributylstannyl)dodeca-2,8-dien-6-ol (**3**)

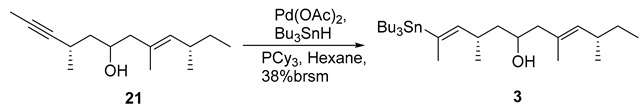



A 25 mL round-bottom flask was charged with Pd(OAc)_2_ (17.3 mg, 0.077 mmol, 0.1 equiv.) and PCy_3_ (53.9 mg, 0.19 mmol, 0.25 equiv.), and dissolved in degassed anhydrous hexane (9 mL). The reaction mixture was purged with argon for 5 min, and stirred for 1 h before a solution of compound **21** (171 mg, 0.77 mmol, 1.0 equiv.) in degassed anhydrous hexane (1 mL). After being stirred for 1 h, Bu_3_SnH (2.1 mL, 7.7 mmol, 10.0 equiv.) was added, and the resulting mixture was stirred for 72 h. The solvent was removed in vacuo and the residue was by flash chromatography on silica gel (4% EtOAc/hexanes, neutral Al_2_O_3_) to give compound **3** as a colorless oil (150 mg, 38% brsm).

**R_f_** = 0.70 (10% EtOAc/hexanes), UV and PMA stain.
${\left[\alpha \right]}_{D}^{20}$ = +25.8 (*c* 0.10, CHCl_3_);
**HRMS** (ESI, *m/z*) for C_27_H_54_OSnNa^+^ [M+Na]^+^: calcd. 537.3089; found: 537.3093.
**^1^H NMR** (400 MHz, CDCl_3_) δ 5.21 (dq, *J* = 9.1, 1.9 Hz, 1H), 5.03–4.96 (m, 1H), 3.67–3.56 (m, 1H), 2.99–2.85 (m, 1H), 2.34–2.19 (m, 1H), 2.13 (dd, *J* = 13.1, 4.4 Hz, 1H), 2.02 (ddd, *J* = 13.2, 8.8, 0.9 Hz, 1H), 1.87 (d, *J* = 1.8 Hz, 3H), 1.66–1.55 (m, 2H),1.60 (d, *J* = 1.4 Hz, 3H), 1.54–1.38 (m, 7H), 1.37–1.16 (m, 10H), 0.98–0.75 (m, 21H).
**^13^C NMR** (101 MHz, CDCl_3_) δ 147.2, 136.6, 135.6, 130.7, 67.0, 48.9, 44.9, 34.3, 30.5, 29.3, 29.1, 27.5, 21.9, 21.3, 19.4, 16.4, 13.9, 12.1, 9.2.

6-((2*S*,4*E*,6*E*,8*S*,12*E*,14*S*)-10-hydroxy-4,6,8,12,14-pentamethylhexadeca-4,6,12-trien-2-yl)-4-(methoxymethoxy)-3,5-dimethyl-2H-pyran-2-one (**22**)

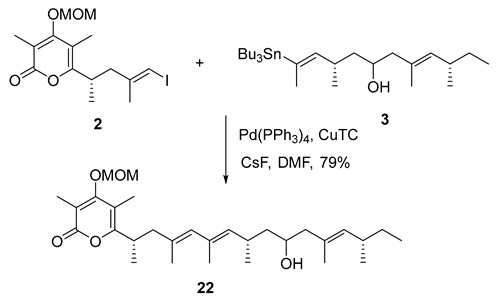



A 25 mL round-bottom flask was charged with compound **2** (66 mg, 0.168 mmol, 1.0 equiv.) and compound **3** (108 mg, 0.210 mmol, 1.25 equiv.) and dissolved in degassed anhydrous DMF (3.4 mL, 0.05 M). The reaction mixture was purged with argon for 5 min, before Pd(PPh_3_)_4_ (19.4 mg, 0.0168 mmol, 0.1 equiv.), CuTC (64 mg, 0.330 mmol, 1.97 equiv.) and CsF (52 mg, 0.342 mmol, 2.03 equiv.) were added sequentially. The resulting mixture was stirred for 48 h before it was quenched by adding aqueous saturated NH_4_Cl (2 mL). The organic layer was separated and the aqueous phase was extracted with EtOAc (3 × 6 mL). The combined organic phases were washed with brine (1 mL), and dried over anhydrous Na_2_SO_4_. The solvent was removed in vacuo and the crude product was purified by flash chromatography on silica gel (33.3% EtOAc/hexanes) to give compound **22** as a colorless oil (64 mg, 79%).

**R_f_** = 0.40 (33% EtOAc/hexanes), UV and PMA stain.
${\left[\alpha \right]}_{D}^{20}$ = +85.4 (*c* 0.50, CHCl_3_);
**HRMS** (ESI, *m/z*) for C_30_H_48_O_5_Na^+^ [M+Na]^+^: calcd. 511.3394; found: 511.3379.
**^1^H NMR** (400 MHz, CDCl_3_) δ 5.56 (s, 1H), 5.02 (s, 2H), 4.98 (dd, *J* = 9.4, 1.7 Hz, 1H), 4.90–4.82 (m, 1H), 3.65–3.55 (m, 1H), 3.56 (s, 3H), 3.13–3.00 (m, 1H), 2.79–2.63 (m, 1H), 2.37 (ddd, *J* = 13.3, 8.2, 1.0 Hz, 1H), 2.32–2.15 (m, 2H), 2.12 (dd, *J* = 13.1, 4.1 Hz, 1H), 2.03–1.97 (m,1H), 2.01 (s, 3H), 1.94 (s, 3H), 1.70 (d, *J* = 1.4 Hz, 3H), 1.65 (d, *J* = 1.4 Hz, 3H), 1.59 (d, *J* = 1.4 Hz, 3H), 1.49–1.21 (m, 4H), 1.20 (d, *J* = 6.8 Hz, 3H), 0.95 (d, *J* = 6.7 Hz, 3H), 0.91 (d, *J* = 6.6 Hz, 3H), 0.82 (t, *J* = 7.4 Hz, 3H).
**^13^C NMR** (101 MHz, CDCl_3_) δ 166.5, 166.0, 161.7, 135.7, 135.3, 132.2, 131.9, 131.7, 130.6, 109.8, 108.7, 99.1, 66.8, 57.9, 48.9, 45.2, 45.1, 34.3, 33.8, 30.5, 29.6, 21.9, 21.3, 17.9, 17.2, 16.5, 12.1, 11.0, 10.5.

4-(methoxymethoxy)-3,5-dimethyl-6-((2*S*,4*E*,6*E*,8*S*,12*E*,14*S*)-4,6,8,12,14-pentamethylhexadeca-4,6,12 -trien-2-yl)-2H-pyran-2-one (**23**)

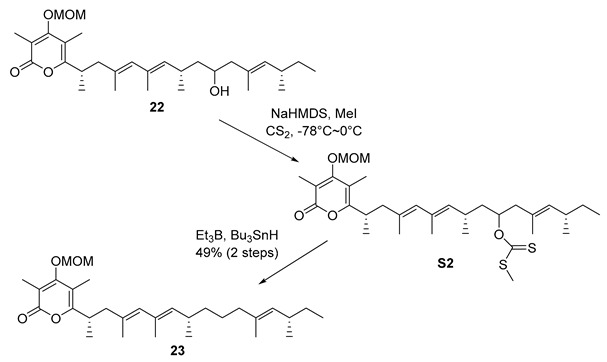



To a cooled (−78 °C) stirred solution of compound **22** (40 mg, 0.0819 mmol, 1.0 equiv.) in anhydrous CS_2_ (8.0 mL, 0.01 M), NaHMDS (61 μL, 0.123 mmol, 2.0 M in THF, 1.5 equiv.) was added. After stirring for 1 h, MeI (77 μL, 1.23 mmol, 15.0 equiv.) was added dropwise. The reaction mixture was allowed to warm to 0 °C and stirred for 3 h, at which point the starting material had been consumed, as verified by TLC analysis; the reaction was quenched by adding aqueous saturated NaHCO_3_ (2 mL) slowly at 0 °C. The organic layer was separated and the aqueous phase was extracted with EA (3 × 5 mL). The combined organic phases were washed with brine (1 mL), and then dried over anhydrous Na_2_SO_4_. The solvent was removed in vacuo at 0 °C and the crude product was purified by flash chromatography on silica gel (25% EtOAc/hexanes) to give the intermediate compound **S2** as a colorless oil.

**R_f_** = 0.50 (25% EtOAc/hexanes), UV and PMA stain.
${\left[\alpha \right]}_{D}^{20}$ = +61.2 (*c* 0.50, CHCl_3_);
**HRMS** (ESI, *m/z*) for C_32_H_50_O_5_S_2_Na^+^ [M+Na]^+^: calcd. 601.2992; found: 601.2990.
**^1^H NMR** (400 MHz, CDCl_3_) δ 5.74–5.63 (m, 1H), 5.52 (s, 1H), 5.02 (s, 2H), 4.92 (d, *J* = 9.3 Hz, 1H), 4.82–4.74 (m, 1H), 3.56 (s, 3H), 3.12–2.98 (m, 1H), 2.61–2.43 (m, 2H), 2.51 (s, 3H), 2.35 (dd, *J* = 13.3, 8.0 Hz, 1H), 2.28–2.11 (m, 3H), 2.01 (s, 3H), 1.93 (s, 3H), 1.81–1.70 (m, 1H), 1.69 (d, *J* = 1.4 Hz, 3H), 1.61 (d, *J* = 1.4 Hz, 3H), 1.59–1.47 (m, 1H), 1.54 (d, *J* = 1.4 Hz, 3H), 1.40–1.09 (m, 2H), 1.18 (d, *J* = 6.8 Hz, 3H), 0.94 (d, *J* = 6.7 Hz, 3H), 0.87 (d, *J* = 6.6 Hz, 3H), 0.81 (t, *J* = 7.4 Hz, 3H).
**^13^C NMR** (101 MHz, CDCl_3_) δ 215.2, 166.5, 166.0, 161.7, 135.6, 134.2, 132.4, 132.4, 131.5, 129.2, 109.8, 108.7, 99.1, 82.1, 57.9, 45.2, 44.5, 41.1, 34.3, 33.8, 30.5, 29.5, 21.6, 20.7, 18.9, 18.0, 17.9, 17.2, 16.7, 12.2, 11.0, 10.5.

To a stirred solution of the above compound **S2** in anhydrous toluene (3 mL, 0.027 M) was added Bu_3_SnH (0.15 mL, 0.573 mmol, 7.0 equiv.) and Et_3_B (98 μL, 0.0983 mmol, 1 M in THF. 1.2 equiv.) sequentially. The reaction mixture was stirred for 12 h, at which point the starting material had been consumed, as verified by TLC analysis; and the reaction was quenched by adding H_2_O (0.5 mL) and KF (71 mg, 1.23 mmol, 15.0 equiv.). The organic layer was separated and the aqueous phase was extracted with EA (3 × 3 mL). The combined organic phases were washed with brine (0.5 mL), and then dried over anhydrous Na_2_SO_4_. The solvent was removed in vacuo and the crude product was purified by flash chromatography on silica gel (20% EtOAc/hexanes) to give compound **23** as a colorless oil (19 mg, 49% for two steps).

**R_f_** = 0.30 (12.5% EtOAc/hexanes), UV and PMA stain.
${\left[\alpha \right]}_{D}^{20}$ = +78.0 (*c* 0.10, CHCl_3_);
**HRMS** (ESI, *m/z*) for C_30_H_48_O_4_Na^+^ [M+Na]^+^: calcd. 495.3445; found: 495.3456.
**^1^H NMR** (400 MHz, CDCl_3_) δ 5.56 (s, 1H), 5.03 (s, 2H), 4.93–4.80 (m, 2H), 3.56 (s, 3H), 3.15–2.97 (m, 1H), 2.42–2.29 (m, 2H), 2.28–2.14 (m, 2H), 2.01 (s, 3H), 1.97–1.87 (m, 2H), 1.94 (s, 3H), 1.70 (d, *J* = 1.4 Hz, 3H), 1.61 (d, *J* = 1.4 Hz, 3H), 1.56 (d, *J* = 1.4 Hz, 3H), 1.42–1.08 (m, 6H), 1.20 (d, *J* = 6.8 Hz, 3H), 0.97–0.85 (m, 6H), 0.82 (t, *J* = 7.4 Hz, 3H).
**^13^C NMR** (101 MHz, CDCl_3_) δ 166.5, 166.0, 161.8, 136.3, 133.8, 132.0, 131.8, 131.4, 130.9, 109.8, 108.7, 99.1, 57.9, 45.3, 40.0, 37.2, 34.1, 33.8, 32.5, 30.7, 26.0, 21.2, 21.2, 17.9, 17.3, 16.2, 12.1, 11.0, 10.5.

4-hydroxy-3,5-dimethyl-6-((2*S*,4*E*,6*E*,8*S*,12*E*,14*S*)-4,6,8,12,14-pentamethylhexadeca-4,6,12-trien-2-yl)-2H-pyran-2-one (**1**)

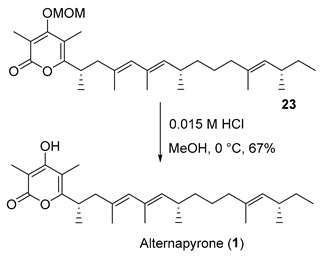



Compound **23** (10 mg, 0.0212 mmol, 1.0 equiv.) was dissolved in HCl/MeOH (21 mL, 0.015 M HCl in MeOH, 0.001 M) and stirred at 0 °C. The reaction was stirred and monitored by TLC. After 12 h, **23** was consumed, as verified by TLC analysis. The solvent was removed in vacuo and the crude product was purified by flash chromatography on silica gel (50% EtOAc/hexanes) to give compound **1** as a colorless oil (6 mg, 67%).

**R_f_** = 0.40 (50% EtOAc/hexanes), UV and PMA stain.
${\left[\alpha \right]}_{D}^{20}$ = +32.0 (*c* 0.10, CHCl_3_);
**HRMS** (ESI, *m/z*) for C_28_H_44_O_3_H^+^ [M+H]^+^: calcd. 429.3363; found: 429.3364.
**^1^H NMR** (400 MHz, Acetone-*d*6) δ 5.58 (s, 1H), 4.94–4.83 (m, 2H), 3.28–3.15 (m, 1H), 2.46–2.37 (m, 1H), 2.33 (ddd, *J* = 13.2, 8.6, 1.0 Hz, 1H), 2.29–2.23 (m, 1H), 2.19 (dd, *J* = 12.9, 6.5 Hz, 1H), 1.96 (s, 3H), 1.92–1.99 (m, 2H), 1.91 (s, 3H), 1.74 (d, *J* = 1.4 Hz, 3H), 1.63 (d, *J* = 1.4 Hz, 3H), 1.57 (d, *J* = 1.4 Hz, 3H), 1.44–1.10 (m, 6H), 1.17 (d, *J* = 6.9 Hz, 3H), 0.92 (d, *J* = 6.7 Hz, 3H), 0.89 (d, *J* = 6.7 Hz, 3H), 0.82 (t, *J* = 7.4 Hz, 3H).
**^13^C NMR** (101 MHz, Acetone-*d*6) δ 165.3, 165.1, 161.5, 136.6, 134.5, 133.3, 132.2, 132.1, 131.9, 106.9, 98.4, 45.9, 40.5, 37.9, 34.8, 34.0, 33.1, 31.3, 26.6, 21.6, 21.4, 18.3, 18.0, 17.4, 16.3, 12.3, 10.0, 9.2.
**Natural alternapyrone^1^:** a colorless oil; ^1^H NMR and ^13^C NMR data (see Table S1); APCI-TOFMS *m*/*z*: calculated for C_28_H_44_O_3_H^+^ [M + H]^+^ 429.3363, found 429.3374

## 4. Conclusions

Alternapyrone consists of an α-pyrone moiety and a 6-alkenyl chain, with its absolute stereochemical configuration previously unknown. The stereochemical configuration was predicted using a biochemistry-guided prediction rule and verified through total synthesis. Key reactions include a retro Diels–Alder reaction to construct the pyrone skeleton, the Kagan–Molander coupling reaction to connect the alkenyl carbon chain, the Stille reaction to form the unstable conjugated diene, and the Barton–McCombie reaction for hydroxyl group removal. The assignment of the absolute configuration of alternapyrone provided strong evidence supporting the applicability of the biochemistry-guided prediction rule in predicting the stereostructures of fungal-reduced polyketide products.

## Data Availability

Copies of ^1^H and ^13^C NMR spectra, as well as associated discussion and structural assignments, are provided in the Supplementary Materials.

## Supplementary Materials

The following supporting information can be downloaded at: https://www.mdpi.com/article/10.3390/molecules30071597/s1, Copies of NMR spectra (^1^H and ^13^C) of **1**–**4**, **7**, **8**, **11**, **12**, **15**, **17**, **20**–**23**, **S1**, **S2**.
