# Supplementary material for: Total Synthesis and Stereochemical Assignment of Alternapyrone
Source: Molecules. 2025 Apr 3;30(7):1597. doi: 10.3390/molecules30071597 (PMC11990127; doi:10.3390/molecules30071597)

Supporting Information

Total Synthesis and Stereochemical Assignment of Alternapyrone

Hui Zhang, Jiaxuan Feng, Di Wang, Dr. Bencan Tang, Chao Xu* and Tao Ye*

Table of Contents:

1. Comparison of NMR Spectra of Natural and Synthetic Alternapyrone S2
2. Table: Comparison of ^{1}H NMR and ^{13}C NMR Data of Alternapyrone S4
3. ECD determination and Calculations S5
4. ^{1}H and ^{13}C NMR Spectra S9

Comparison of NMR Spectra of Natural and Synthetic Alternapyrone

[1]**H NMR** (natural product, 400 MHz, acetone-*d*6)

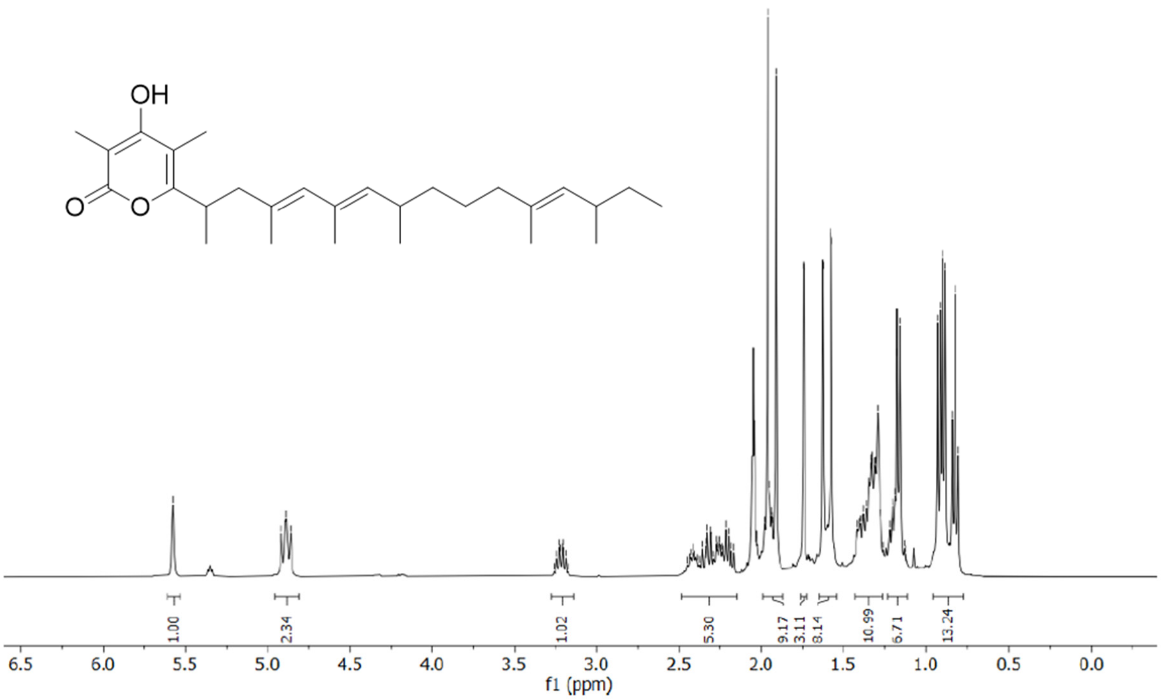

[1]**H NMR** (synthetic product, 400 MHz, acetone-*d*6)

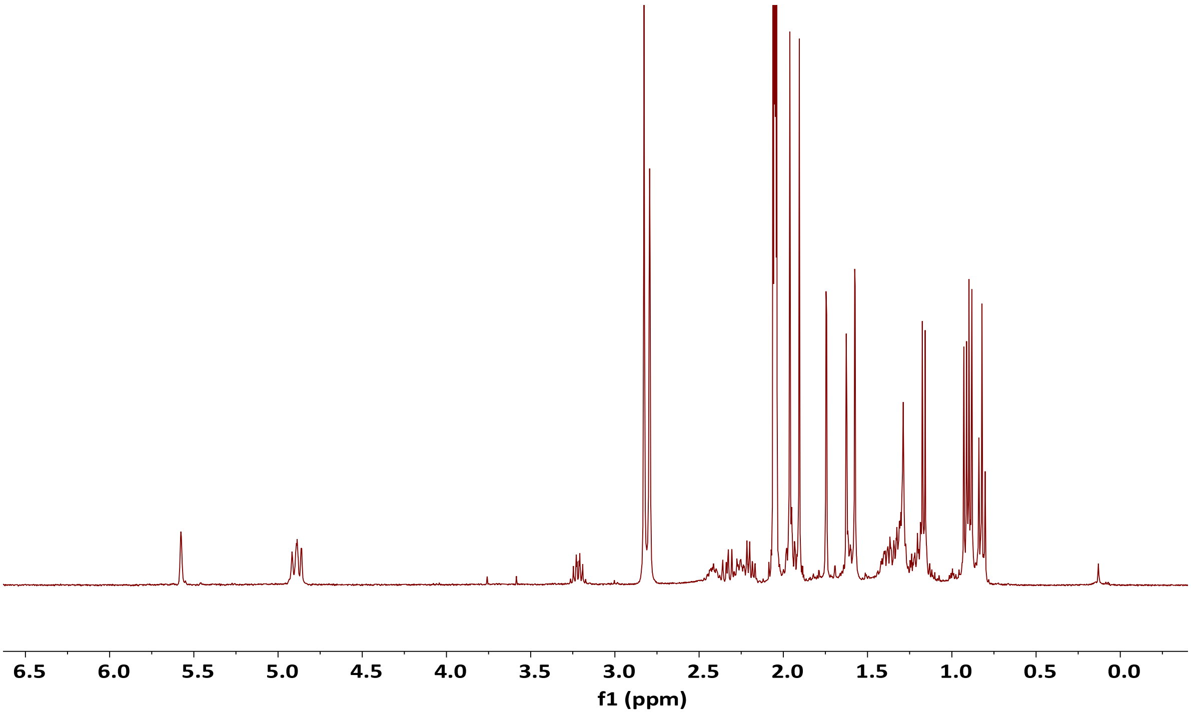

^{13}C NMR (natural product, 100 MHz, acetone-*d6*)

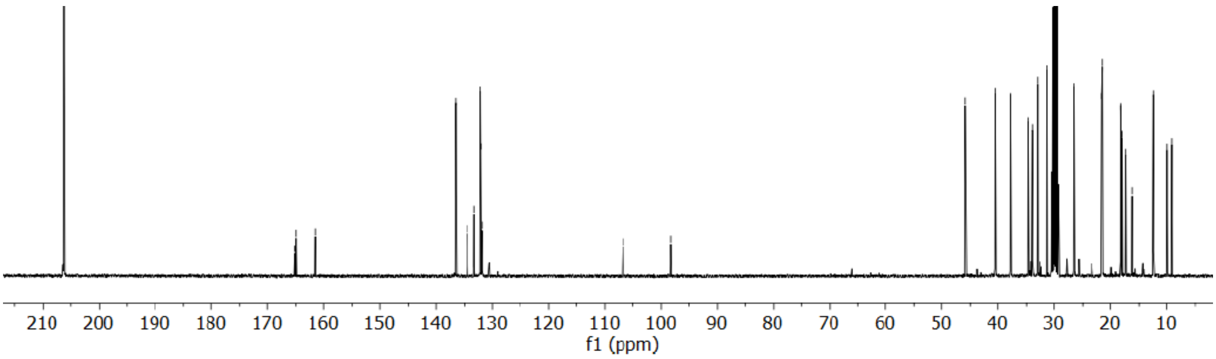

^{13}C NMR (synthetic product, 101 MHz, acetone-*d6*)

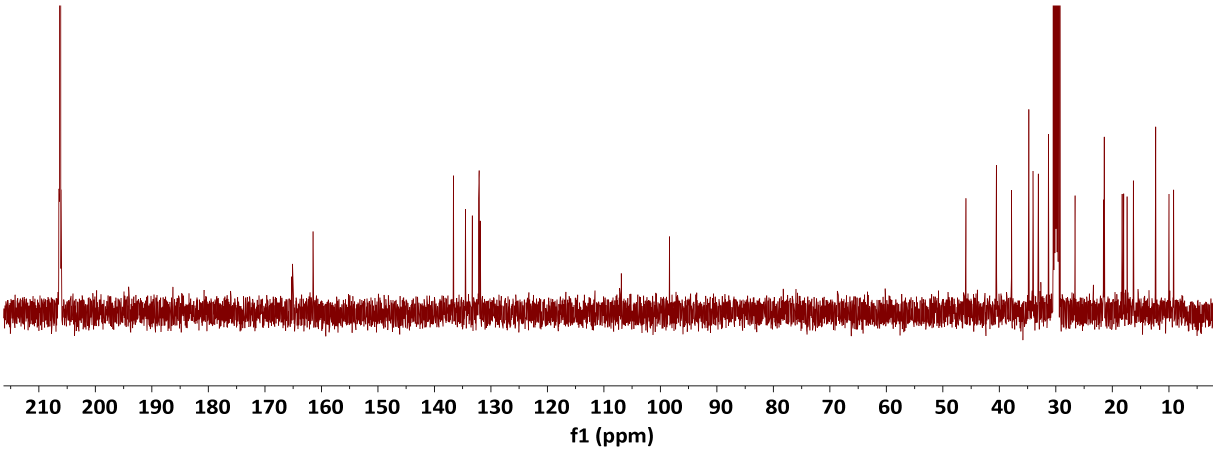

Table S1 Comparison of NMR data of alternapyrone

Position	δH(Nat.)/ ppm (mult., *J*)	δH(Syn.)/ ppm (mult., *J*)	δC(Nat.)/ ppm	δC(Syn.)/ ppm	ΔδC(Syn.-Nat.)/ppm
1			165.2	165.3	0.1
2			98.3	98.4	0.1
3			165.0	165.1	0.1
4			106.8	106.9	0.1
5			161.4	161.5	0.1
6	3.22 (m)	3.23 (m)	34.0	34.0	0.0
7	2.33 (dd, 13.0, 8.3)	2.33 (dd, 13.2, 8.6)	45.9	45.9	0.0
	2.19 (dd, 13.0, 6.5)	2.19 (dd, 13.0, 6.5)			
8			133.2	133.3	0.1
9	5.58 (s)	5.58 (s)	132.1	132.2	0.1
10			131.8	131.9	0.1
11	4.91 (br d, 11.1)	4.90 (m, overlapped)	136.6	136.6	0.0
12	2.39-2.45 (m)	2.37-2.46 (m)	33.0	33.1	0.1
13	1.16-1.22 (m)	1.16-1.24 (m, overlapped)	37.8	37.9	0.1
	1.26-1.34 (m)	1.27-1.34 (m, overlapped)			
14	1.36-1.42 (m)	1.36-1.44 (m, overlapped)	26.6	26.6	0.0
15	1.93-1.98 (m)	1.92-1.99 (m, overlapped)	40.5	40.5	0.0
16			134.4	134.5	0.1
17	4.88 (br d, 10.9)	4.88 (m, overlapped)	132.0	132.1	0.1
18	2.23-2.27 (m)	2.22-2.29 (m)	34.7	34.8	0.1
19	1.12-1.22 (m)	1.13-1.22 (m, overlapped)	31.3	31.3	0.0
	1.27-1.38 (m)	1.27-1.36 (m, overlapped)			
20	0.82 (t, 7.4)	0.82 (t, 7.4)	12.3	12.3	0.0
21	1.91 (s)	1.91 (s)	9.1	9.2	0.1
22	1.96 (s)	1.96 (s)	10.0	10.0	0.0
23	1.17 (d, 7.3)	1.17 (d, 6.8)	18.2	18.3	0.1
24	1.74 (d, 1.4)	1.74 (d, 1.4)	18.0	18.0	0.0
25	1.62 (d, 1.4)	1.63 (d, 1.4)	17.3	17.4	0.1
26	0.92 (d, 6.7)	0.92 (d, 6.6)	21.5	21.6	0.1
27	1.57 (d, 1.4)	1.58 (d, 1.4)	16.2	16.3	0.1
28	0.89 (d, 6.7)	0.89 (d, 6.7)	21.4	21.4	0.0

Comparison of ECD Spectra of Synthetic Alternapyrone and Calculated Spectra for the two possible enantiomers

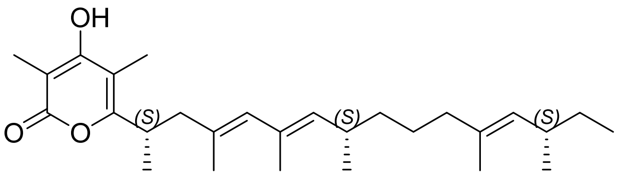

1-*SSS*

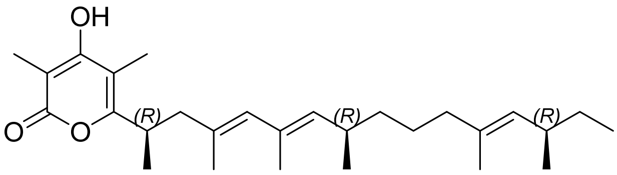

1'-*RRR*

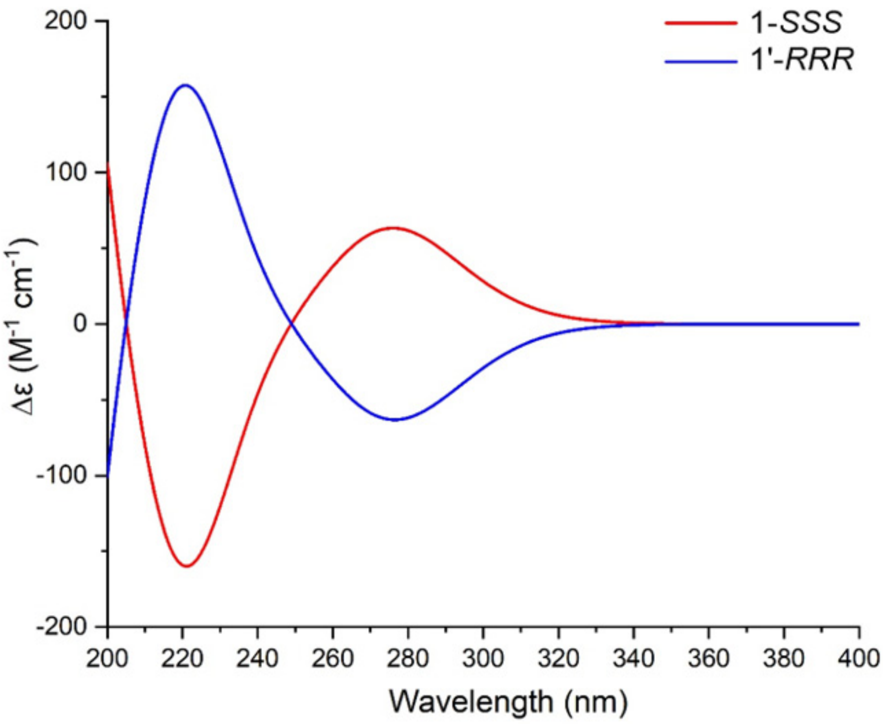

ECD Spectra of Synthetic Alternapyrone: Solvent: methanol; concentration: 0.1 mg/mL

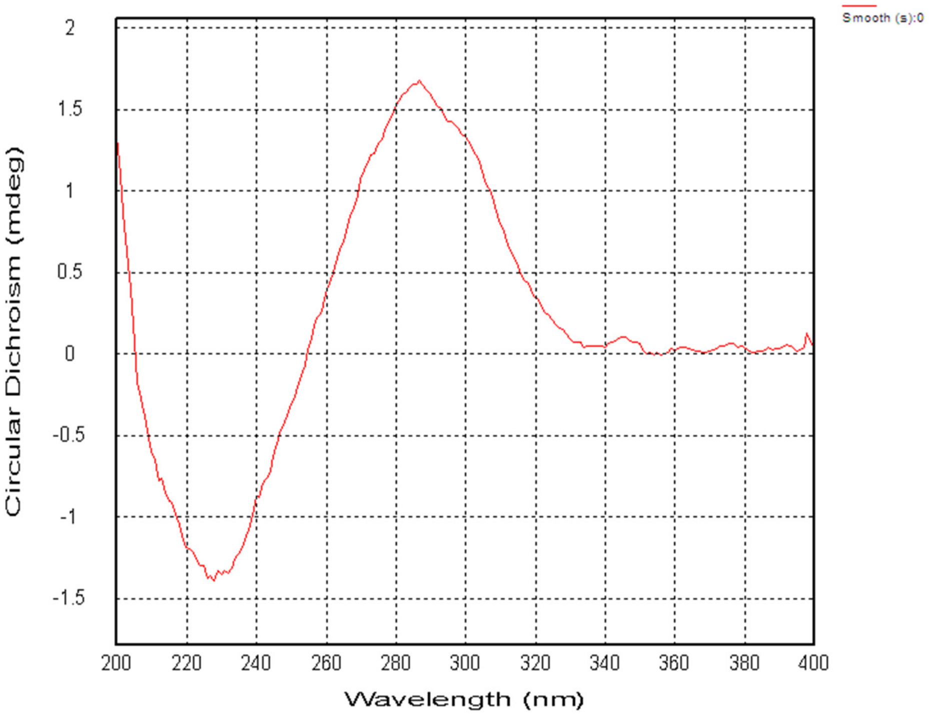

ECD Calculations. All DFT calculations were carried out using Gaussian 16 software package [1]. In general, conformational analyses were carried out *via* random searching in GMMX 3.1 module using the MMFF94 force field with an energy cutoff of 3.0 kcal/mol. Eight lowest energy conformers were selected for the subsequent geometry optimization and vibrational frequency analysis at the PBE0-D3(BJ)/def2-SVP level with the SMD implicit solvent model of methanol. The single point energies for all optimized structures were calculated at the M06-2X-D3/def2-TZVP level with the SMD implicit solvent model of methanol. The oscillator strengths and rotational strengths of the first 30 electronic excitations were calculated using TDDFT methodology at the PBE0-D3(BJ)/def2-TZVP level with the SMD implicit solvent model of methanol. To get the final spectra, the simulated spectra of the conformers were weighted average according to the Boltzmann distribution theory and their relative Gibbs free energy (ΔG). The experimental and simulated spectra were plotted by OriginPro Learning Edition and Multiwfn [2].

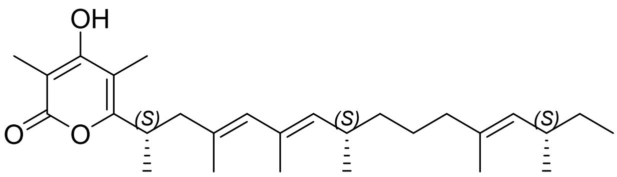

1-*SSS*

Table S2. Gibbs free energies[a] and the equilibrium population[b] of low energy conformers of 1-*SSS*

Conformers	ΔG (kcal/mol)	Percent (%)
**1-*SSS*-1**	1.04	11.32
**1-*SSS*-2**	2.03	2.13
**1-*SSS*-3**	0.85	15.60
**1-*SSS*-4**	3.13	0.33
**1-*SSS*-5**	3.28	0.26
**1-*SSS*-6**	0.00	65.49
**1-*SSS*-7**	2.83	0.55
**1-*SSS*-8**	1.61	4.33

[a] SMD(Methanol)-M06-2X-D3/def2-TZVP, in kcal/mol.

[b] Δ*G* values at 298.15K.

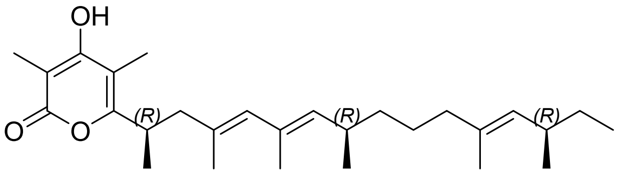

1'-*RRR*

Table S3. Gibbs free energies[a] and the equilibrium population[b] of low energy conformers of 1'-*RRR*

Conformers	ΔG (kcal/mol)	Percent (%)
**1'-*RRR*-1**	0.96	11.40
**1'-*RRR*-2**	1.77	2.91
**1'-*RRR*-3**	0.98	11.03
**1'-*RRR*-4**	0.79	15.19
**1'-*RRR*-5**	3.29	0.22
**1'-*RRR*-6**	0.00	57.64
**1'-*RRR*-7**	2.18	1.45
**1'-*RRR*-8**	3.53	0.15

[a] SMD(Methanol)-M06-2X-D3/def2-TZVP, in kcal/mol.

[b] Δ*G* values at 298.15K.

References

[1] Gaussian 16, Revision A.03, M. J. Frisch, G. W. Trucks, H. B. Schlegel, G. E. Scuseria, M. A. Robb, J. R. Cheeseman, G. Scalmani, V. Barone, G. A. Petersson, H. Nakatsuji, X. Li, M. Caricato, A. V. Marenich, J. Bloino, B. G. Janesko, R. Gomperts, B. Mennucci, H. P. Hratchian, J. V. Ortiz, A. F. Izmaylov, J. L. Sonnenberg, D. Williams-Young, F. Ding, F. Lipparini, F. Egidi, J. Goings, B. Peng, A. Petrone, T. Henderson, D. Ranasinghe, V. G. Zakrzewski, J. Gao, N. Rega, G. Zheng, W. Liang, M. Hada, M. Ehara, K. Toyota, R. Fukuda, J. Hasegawa, M. Ishida, T. Nakajima, Y. Honda, O. Kitao, H. Nakai, T. Vreven, K. Throssell, J. A. Montgomery, Jr., J. E. Peralta, F. Ogliaro, M. J. Bearpark, J. J. Heyd, E. N. Brothers, K. N. Kudin, V. N. Staroverov, T. A. Keith, R. Kobayashi, J. Normand, K. Raghavachari, A. P. Rendell, J. C. Burant, S. S. Iyengar, J. Tomasi, M. Cossi, J. M. Millam, M. Klene, C. Adamo, R. Cammi, J. W. Ochterski, R. L. Martin, K. Morokuma, O. Farkas, J. B. Foresman, and D. J. Fox, Gaussian, Inc., Wallingford CT, 2016.

[2] Lu, T.; Chen, F., Multiwfn: A multifunctional wavefunction analyzer. *J. Comput. Chem.* **2011**, *33*, 580-592. https://doi.org/10.1002/jcc.22885.

^{1}H NMR (400 MHz, $CDCl_3$)

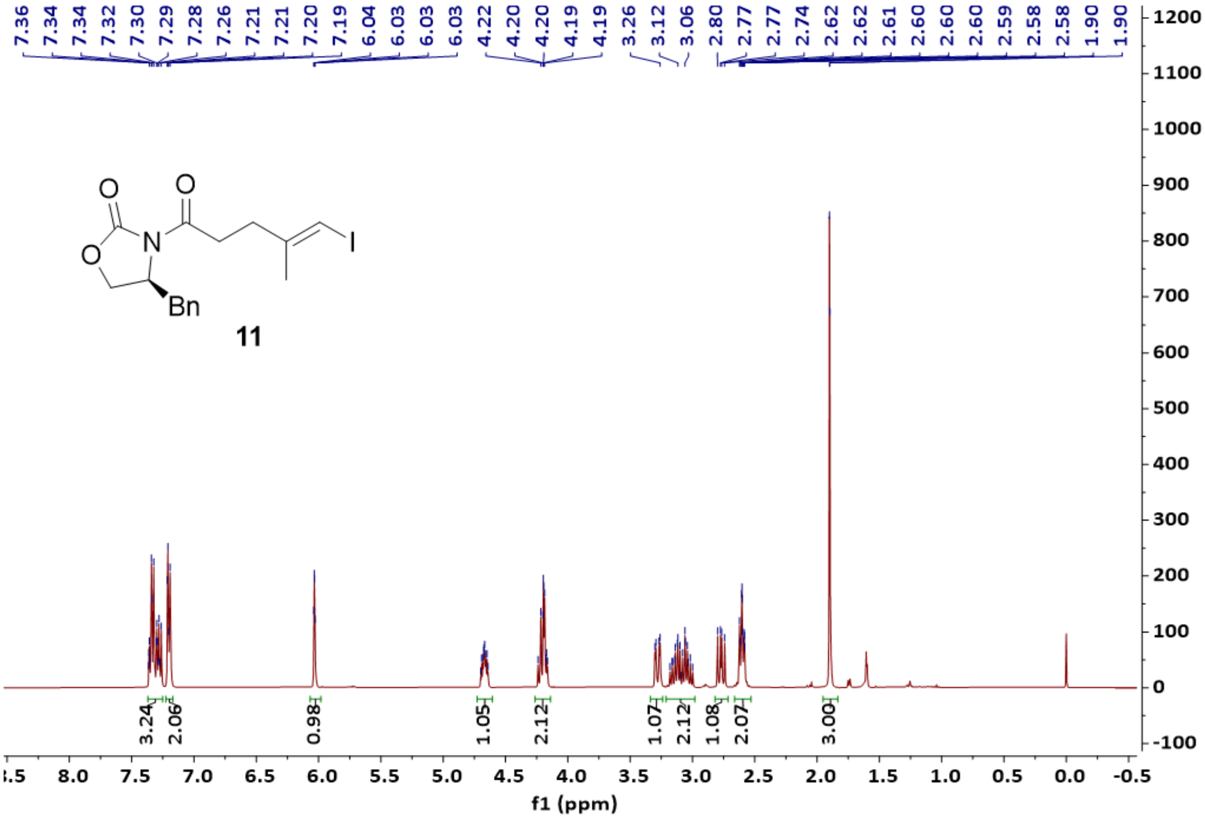

^{13}C NMR (101 MHz, $CDCl_3$)

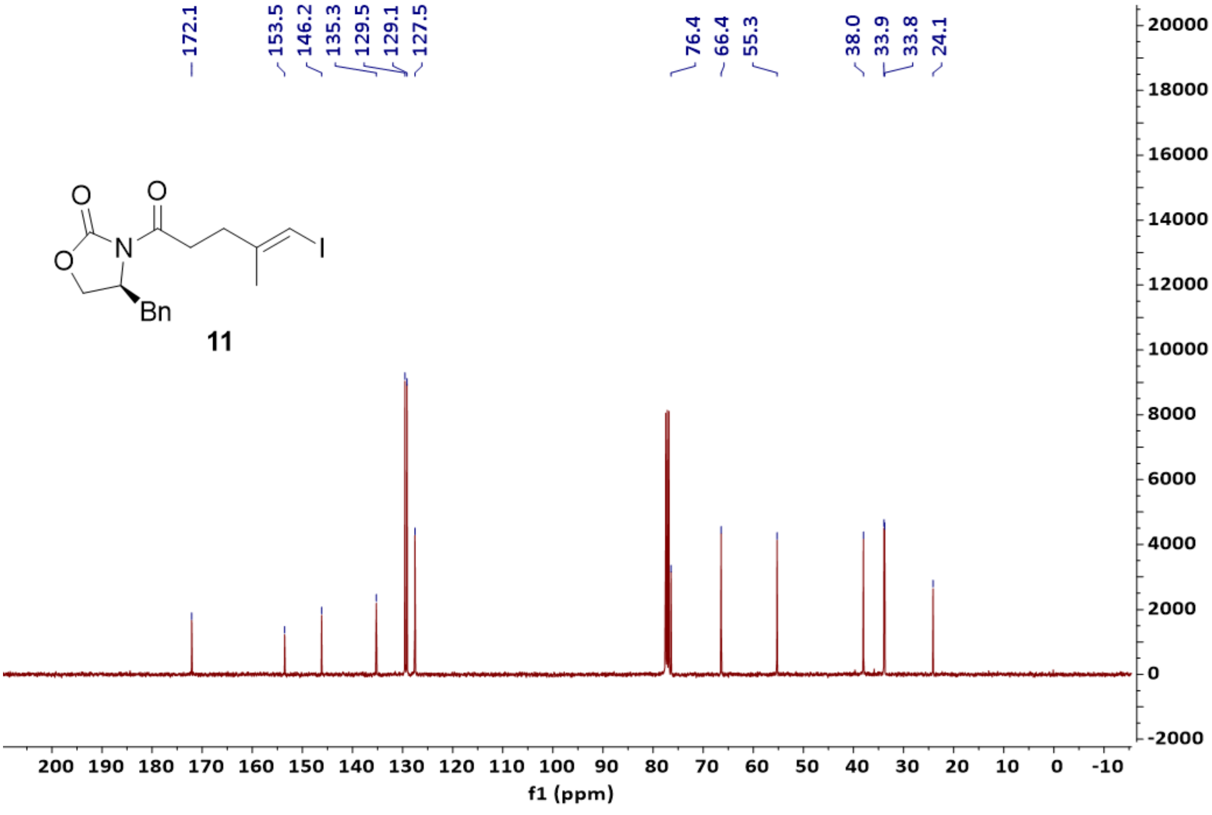

^{1}H NMR (500 MHz, $CDCl_3$)

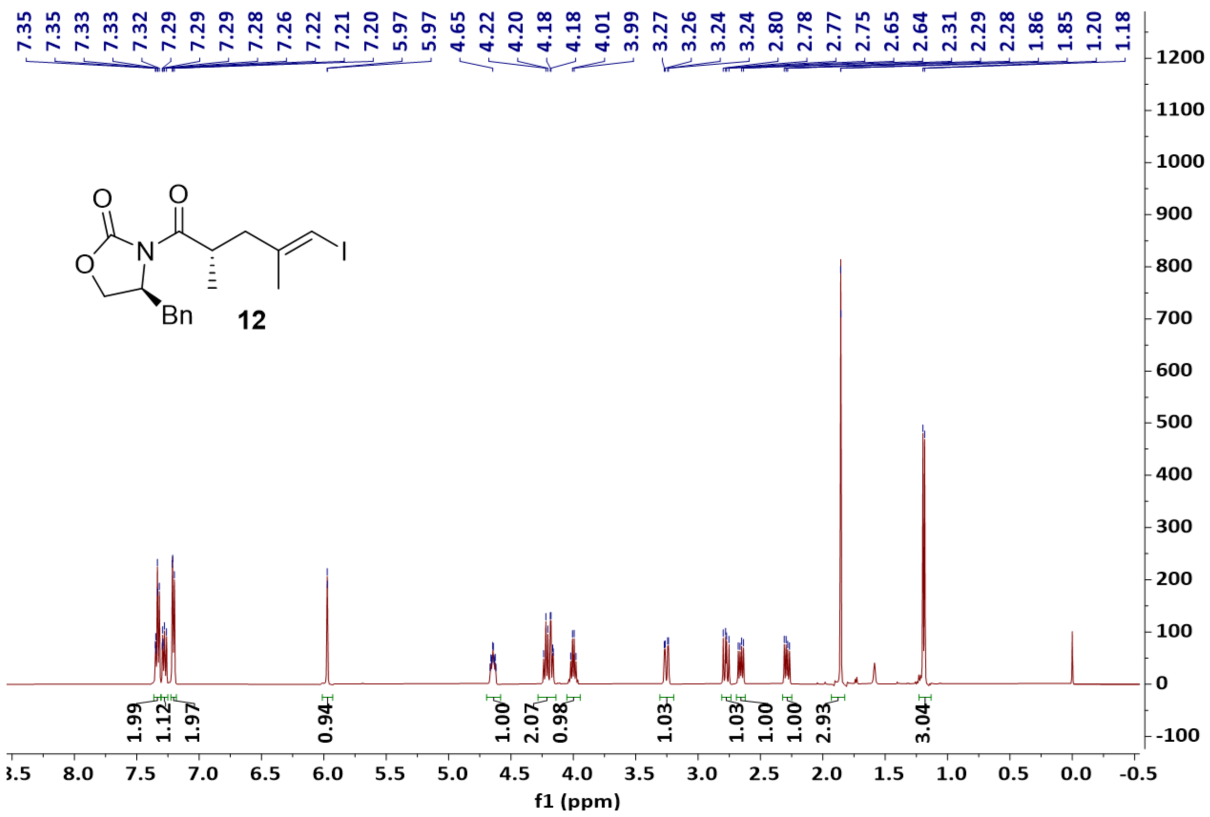

^{13}C NMR (101 MHz, $CDCl_3$)

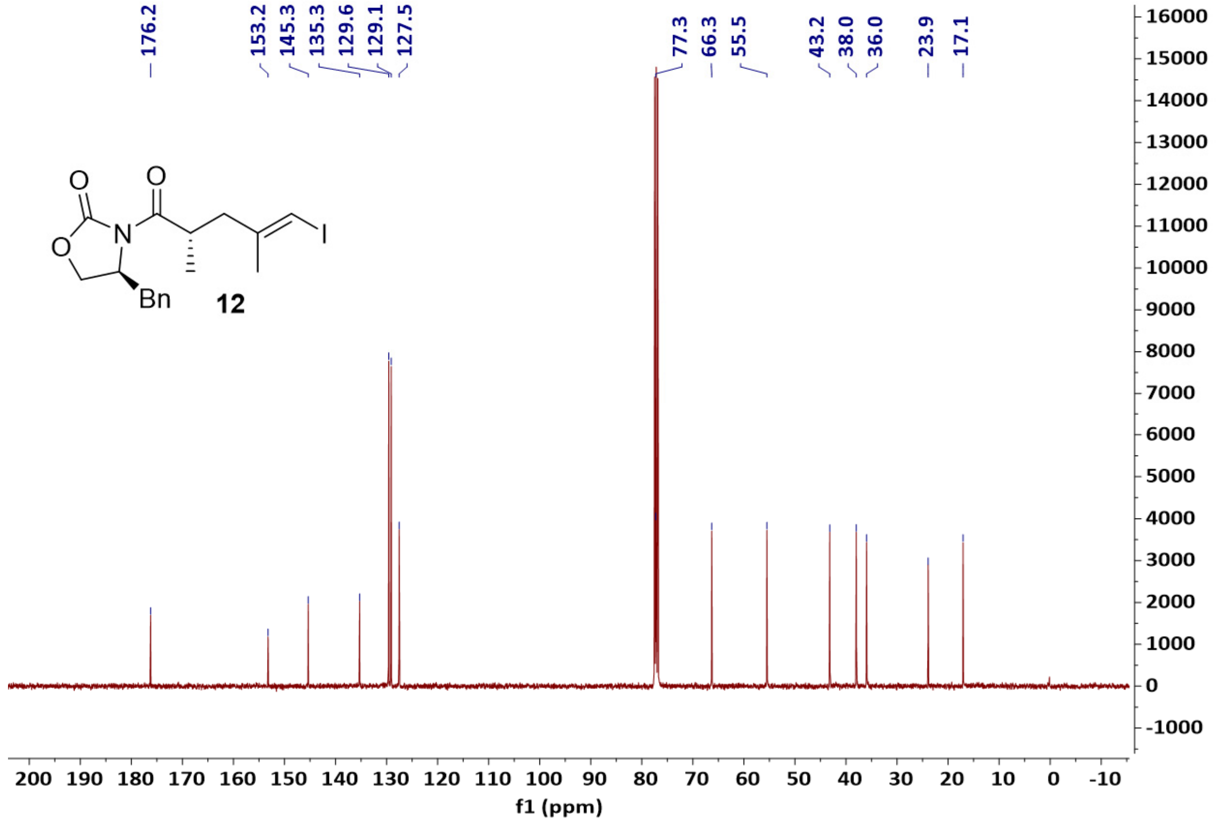

^{1}H NMR (400 MHz, $CDCl_3$)

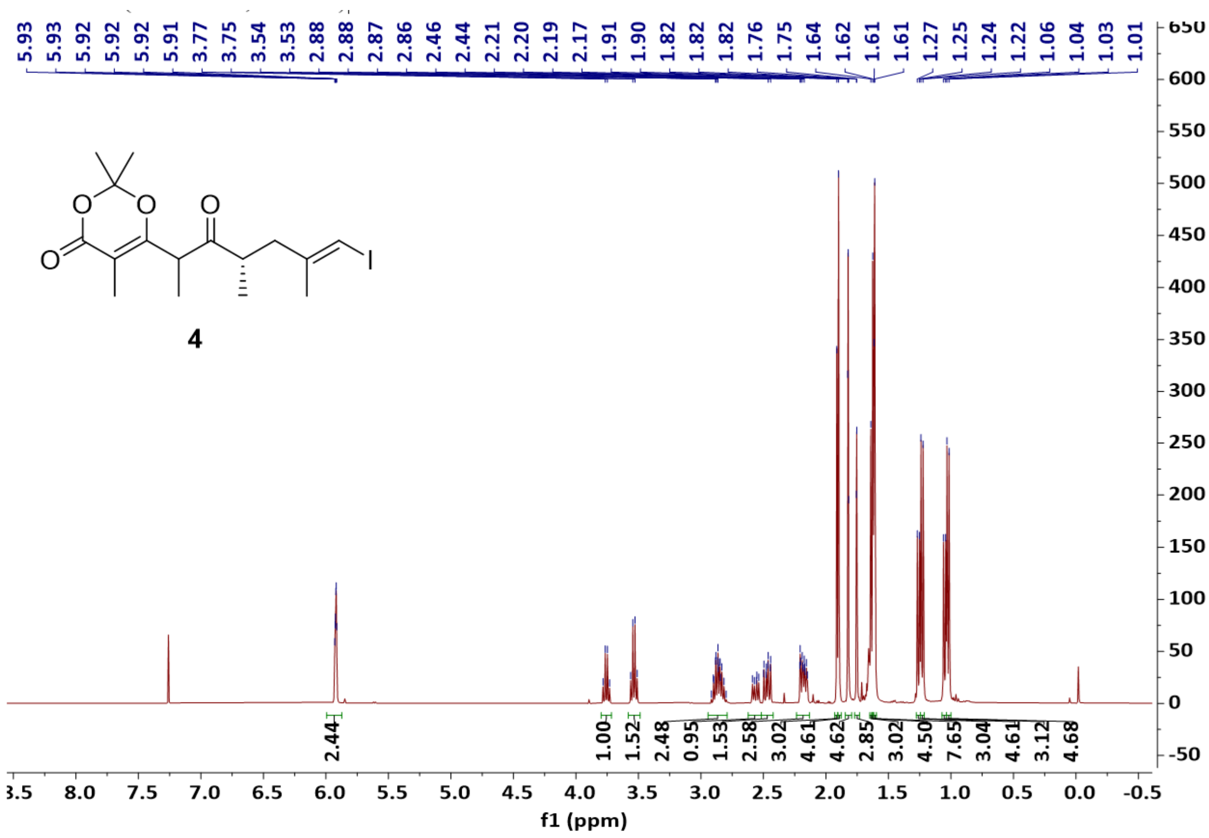

^{13}C NMR (101 MHz, $CDCl_3$)

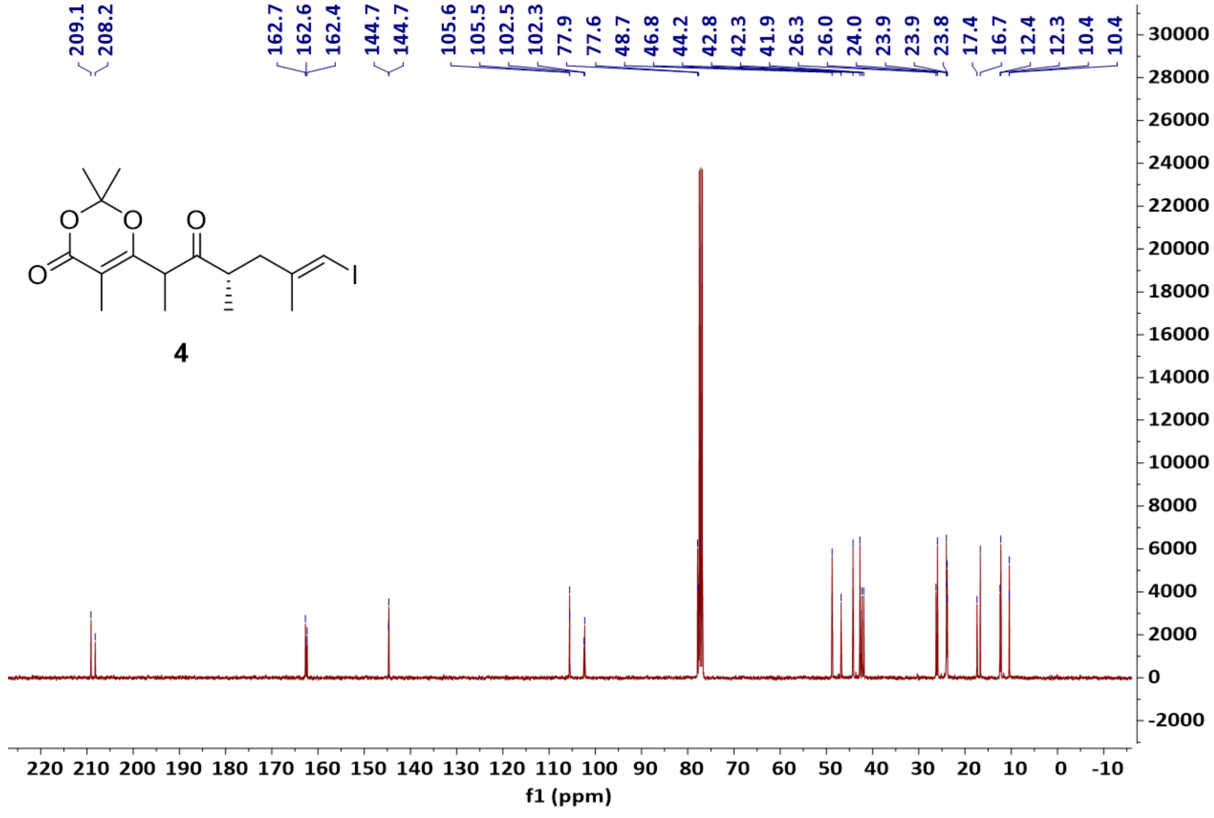

^{1}H NMR (400 MHz, $CDCl_3$)

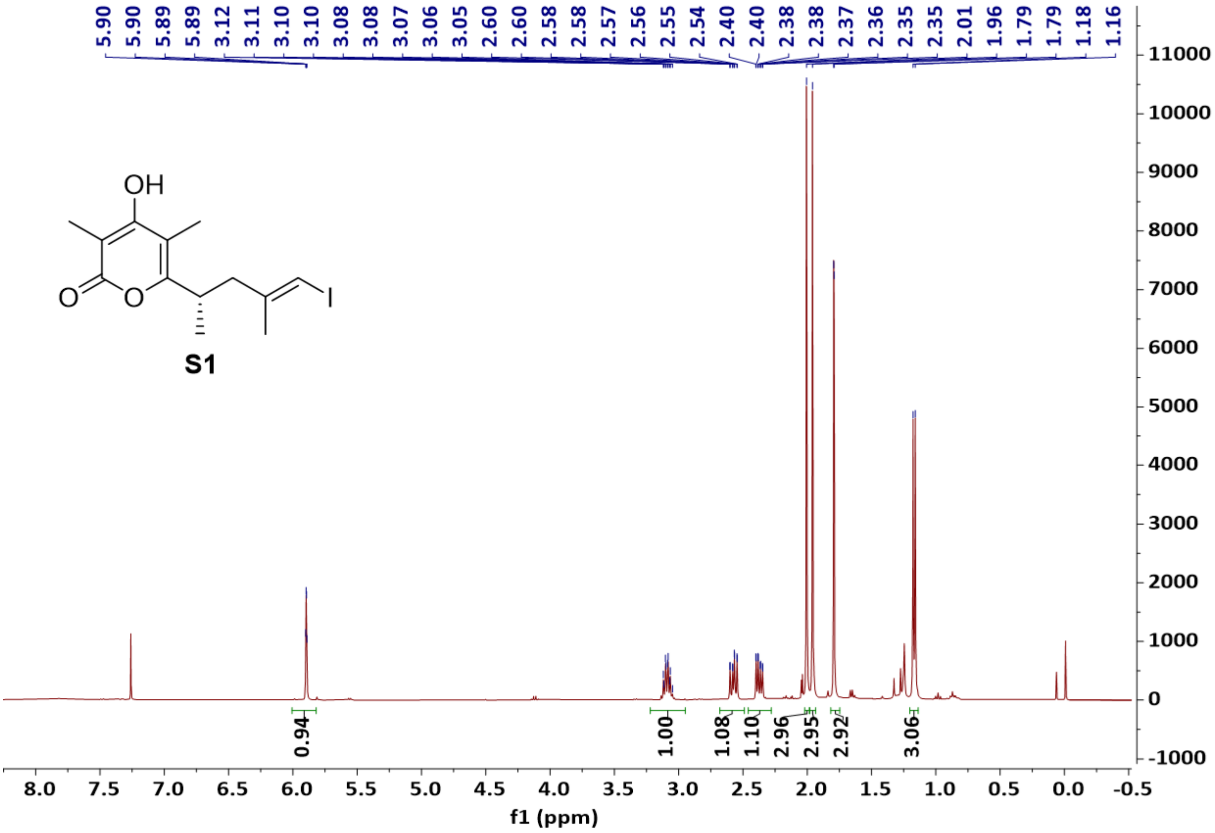

^{13}C NMR (101 MHz, $CDCl_3$)

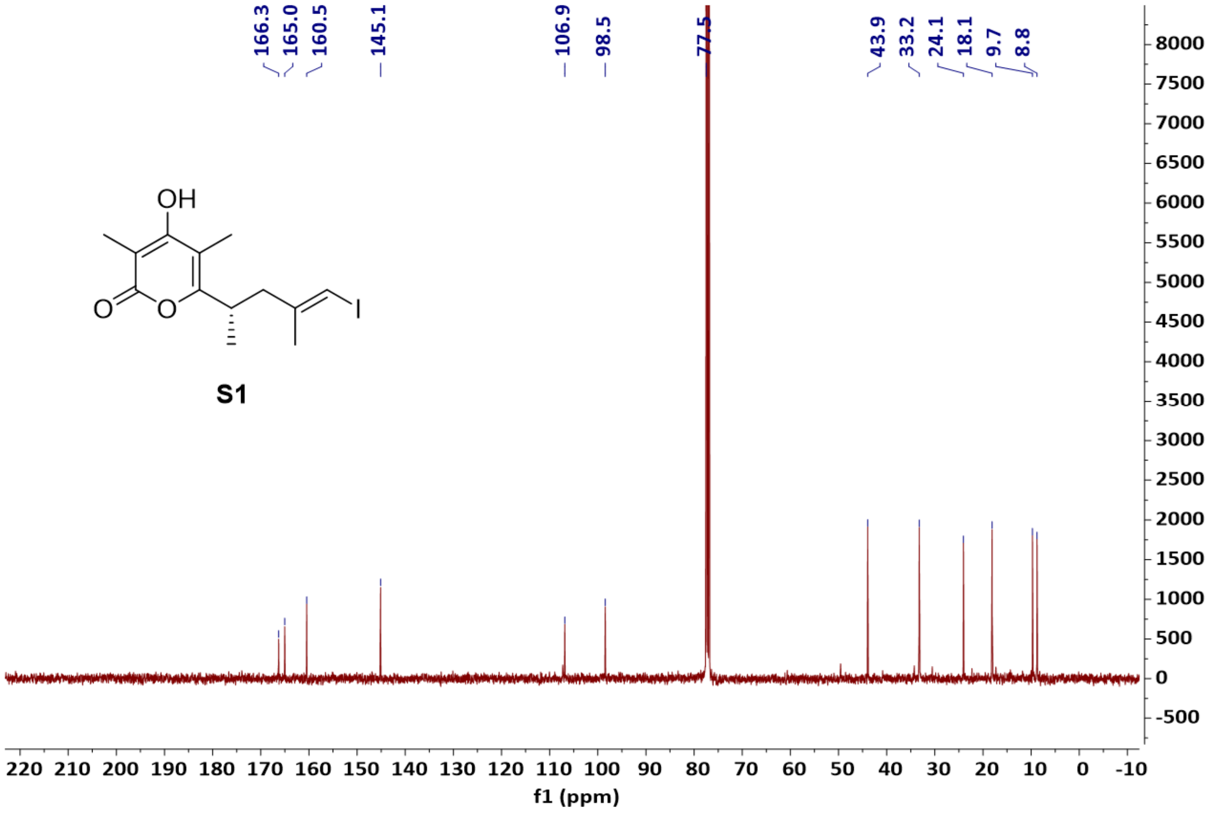

^{1}H NMR (500 MHz, $CDCl_3$)

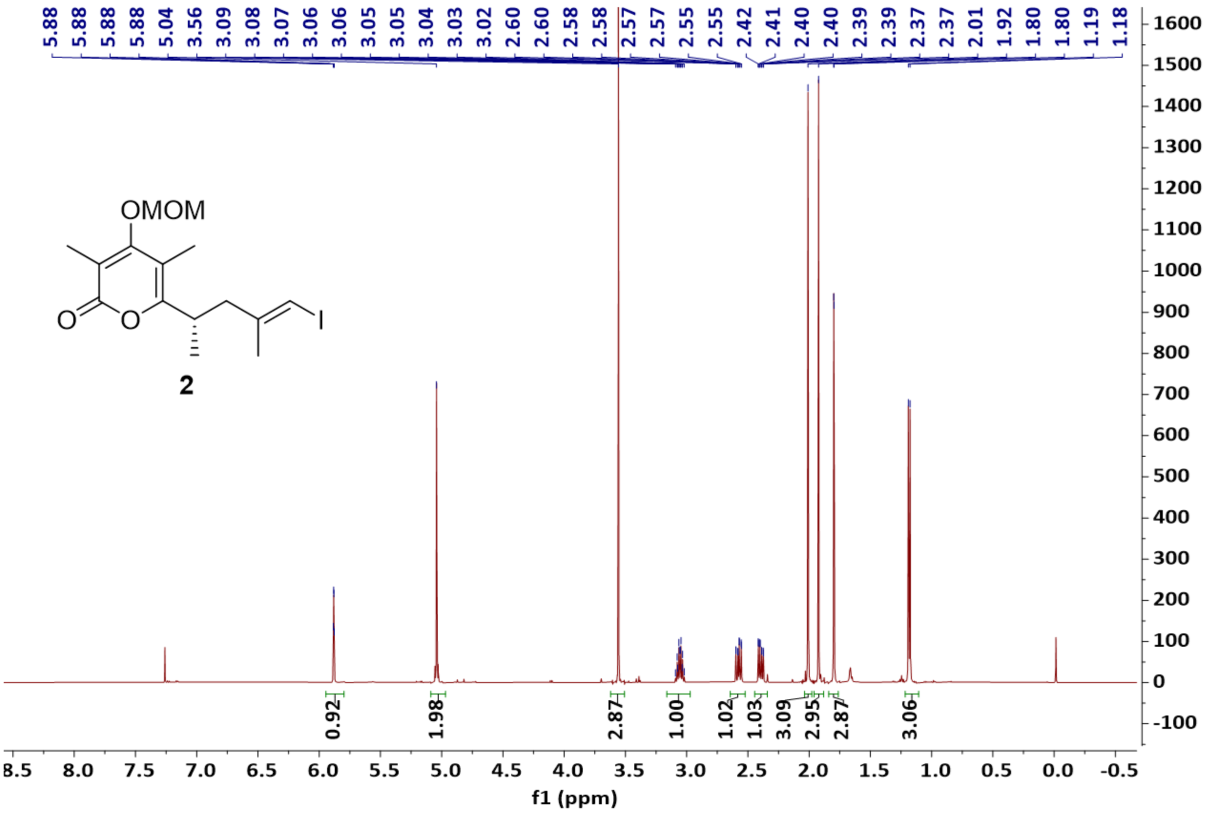

^{13}C NMR (101 MHz, $CDCl_3$)

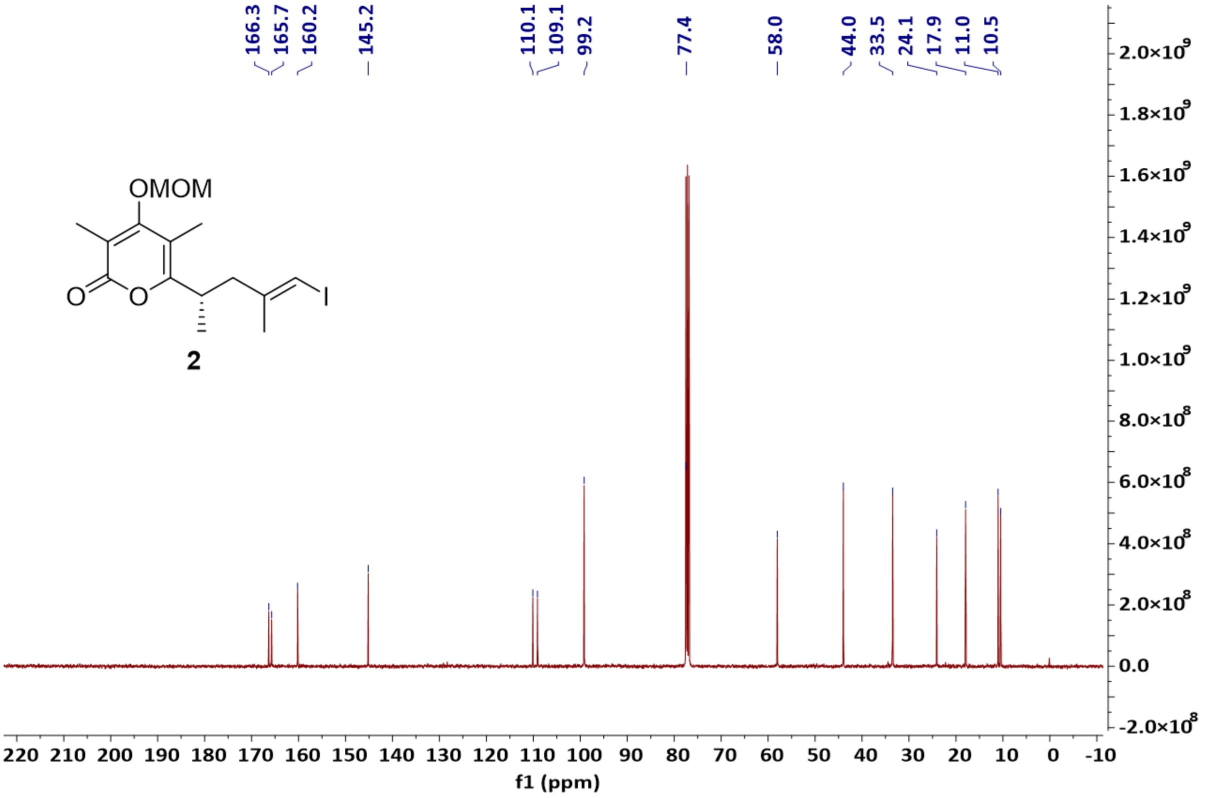

^{1}H NMR (400 MHz, $CDCl_3$)

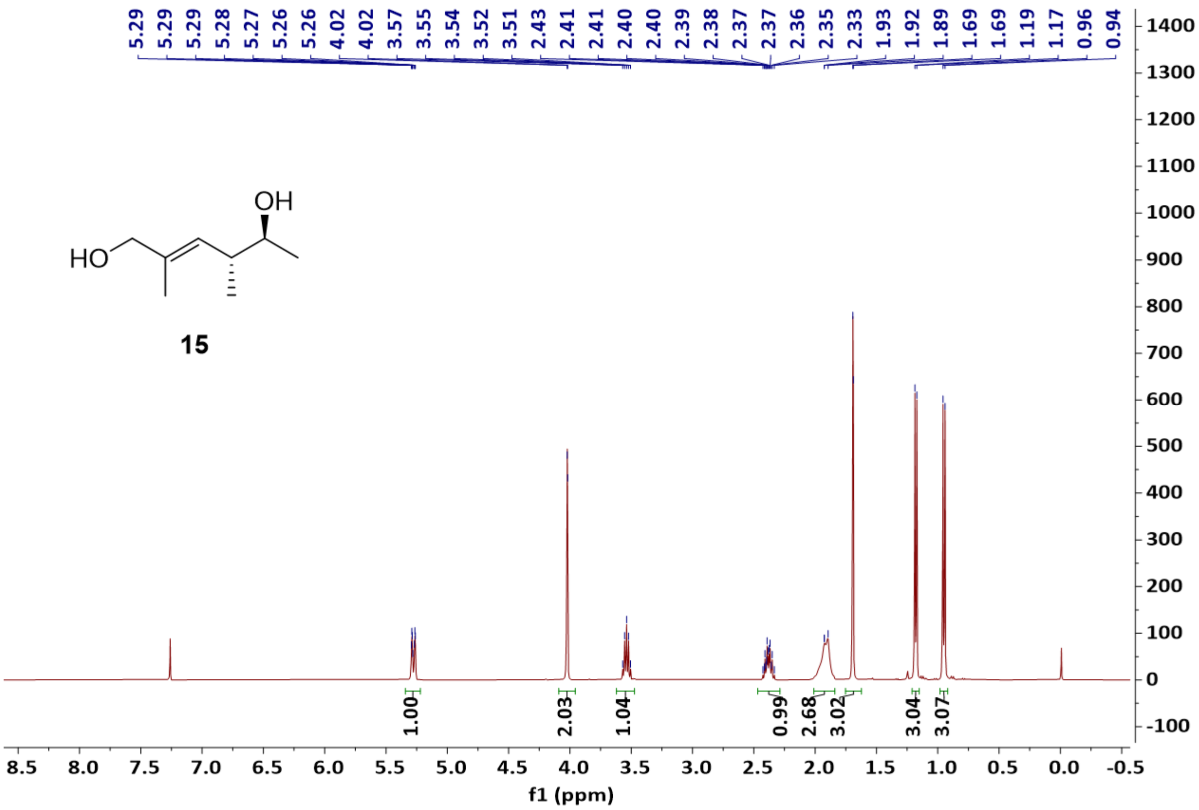

^{13}C NMR (101 MHz, $CDCl_3$)

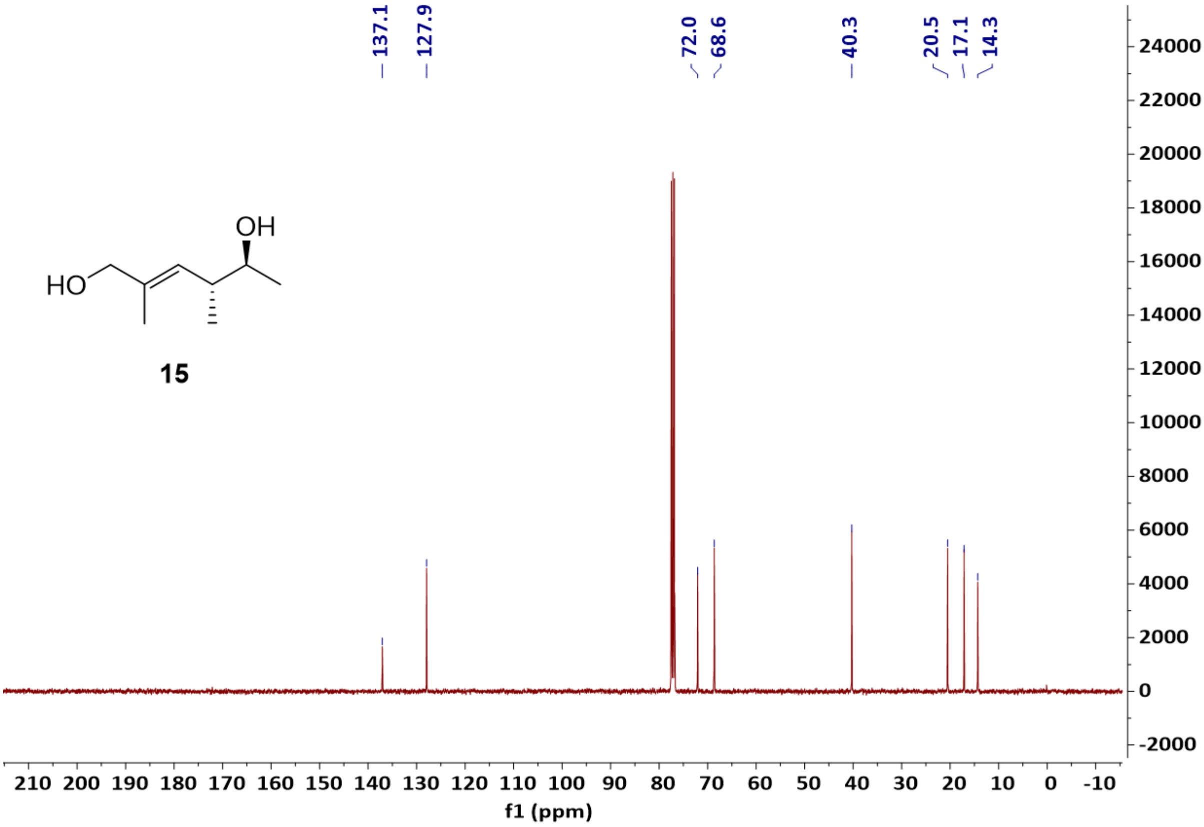

^{1}H NMR (500 MHz, $CDCl_3$)

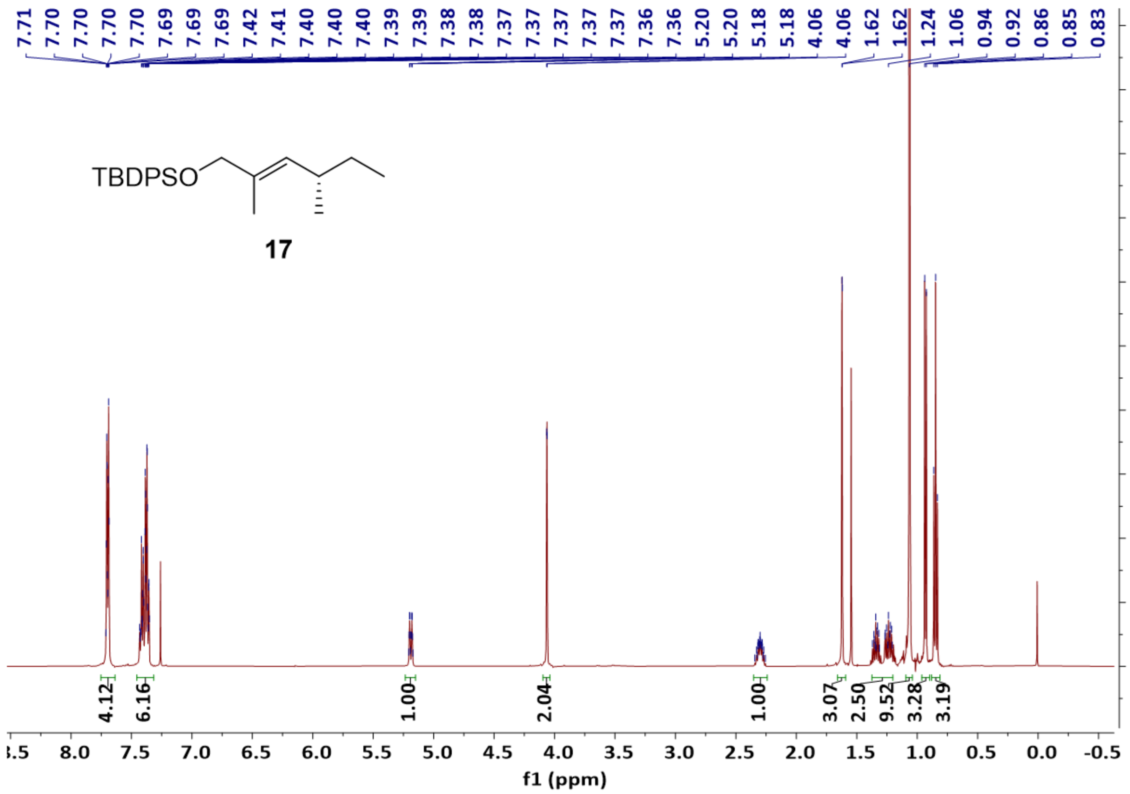

^{13}C NMR (101 MHz, $CDCl_3$)

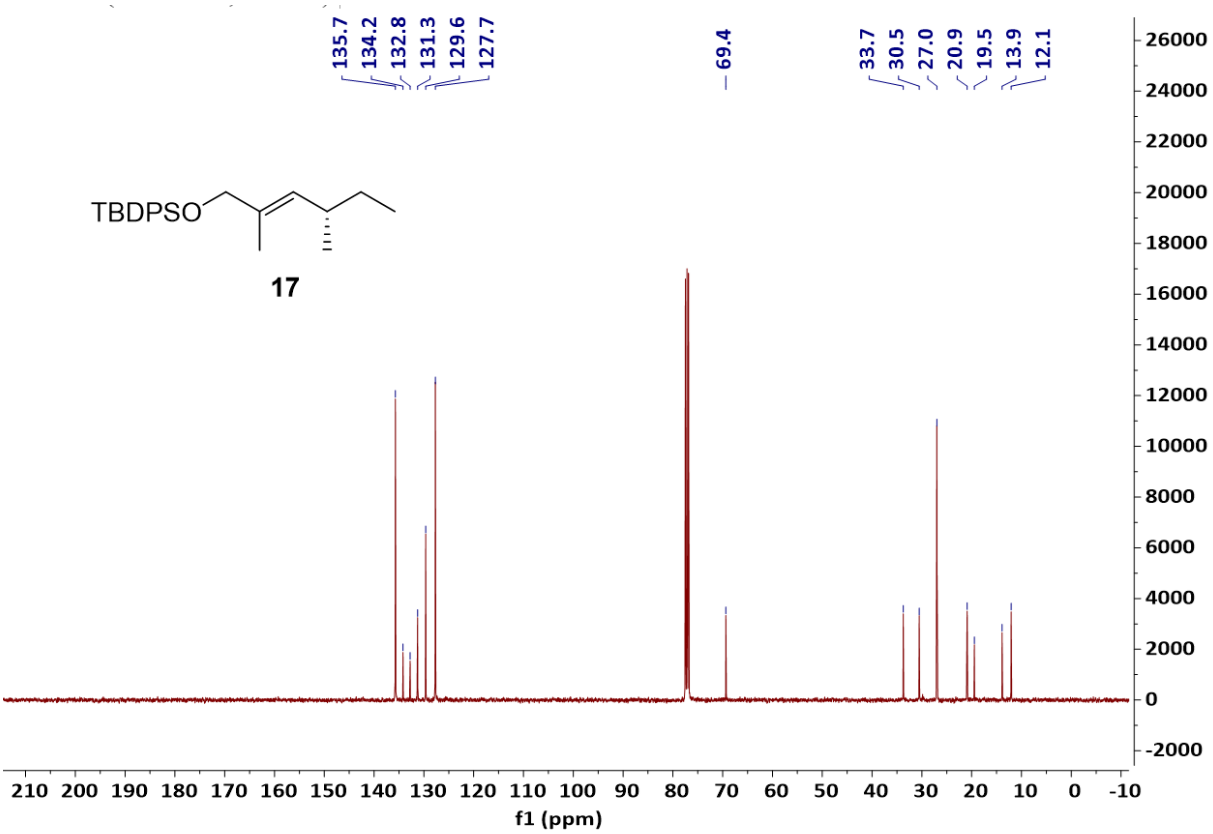

^{1}H NMR (400 MHz, $CDCl_3$)

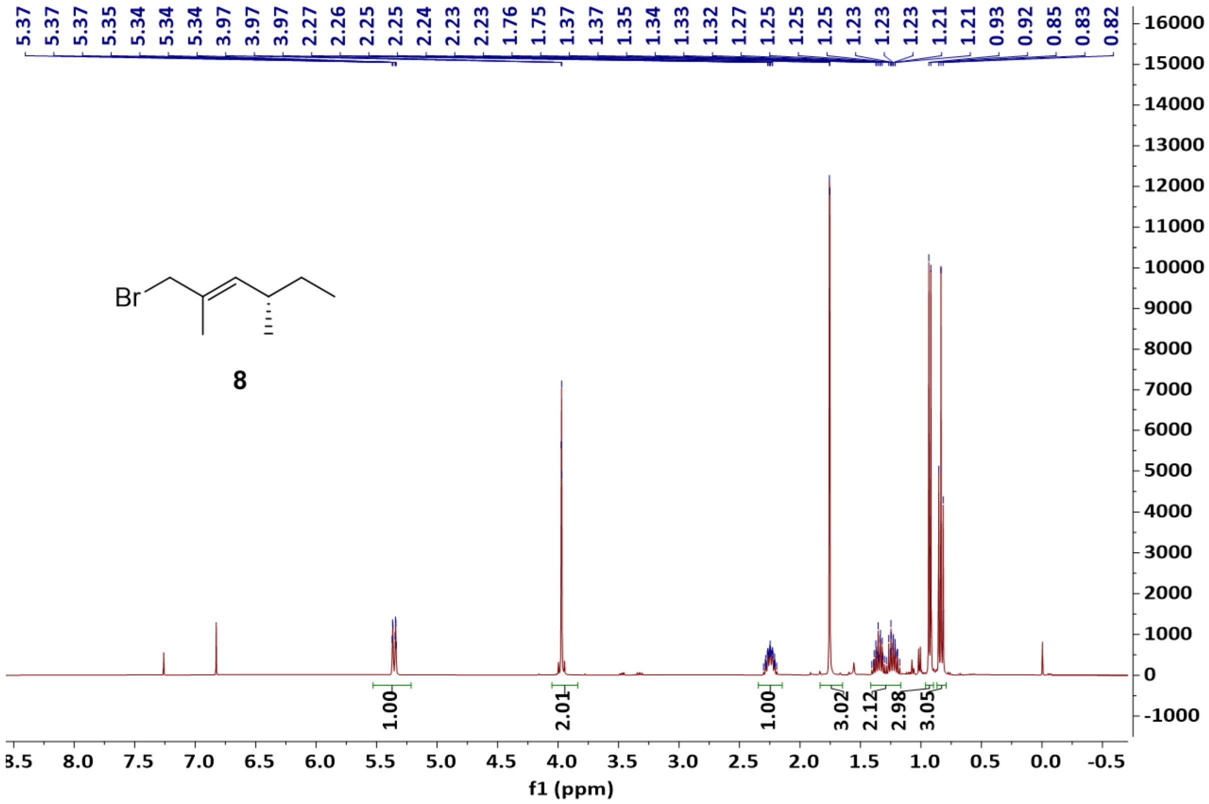

^{13}C NMR (101 MHz, $CDCl_3$)

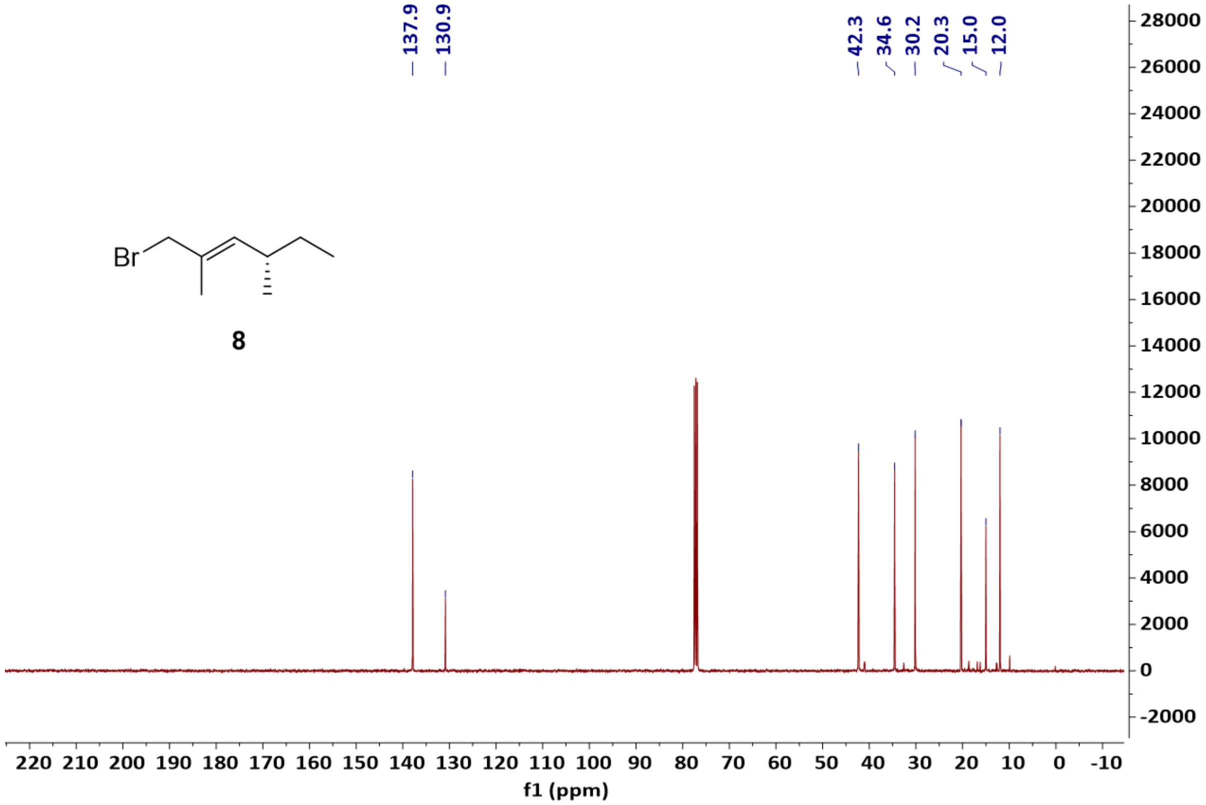

^{1}H NMR (500 MHz, $CDCl_3$)

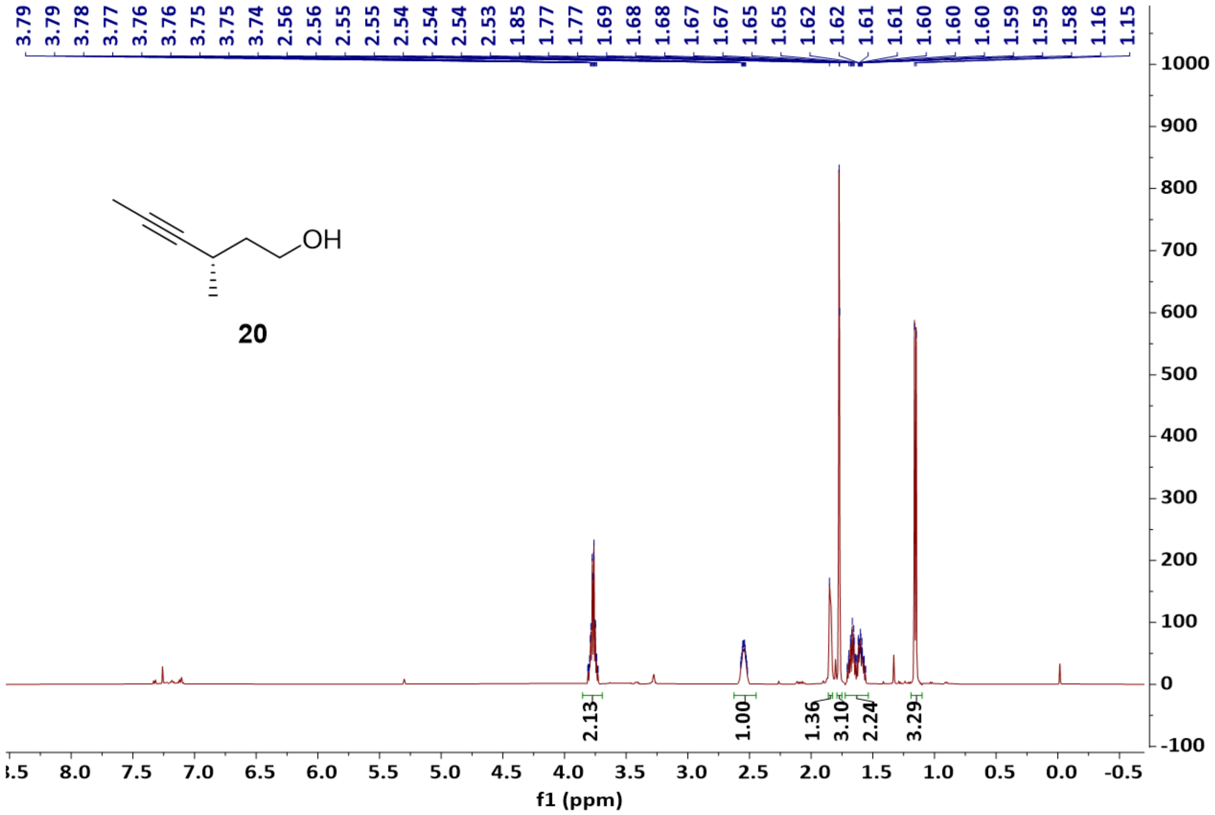

^{13}C NMR (101 MHz, $CDCl_3$)

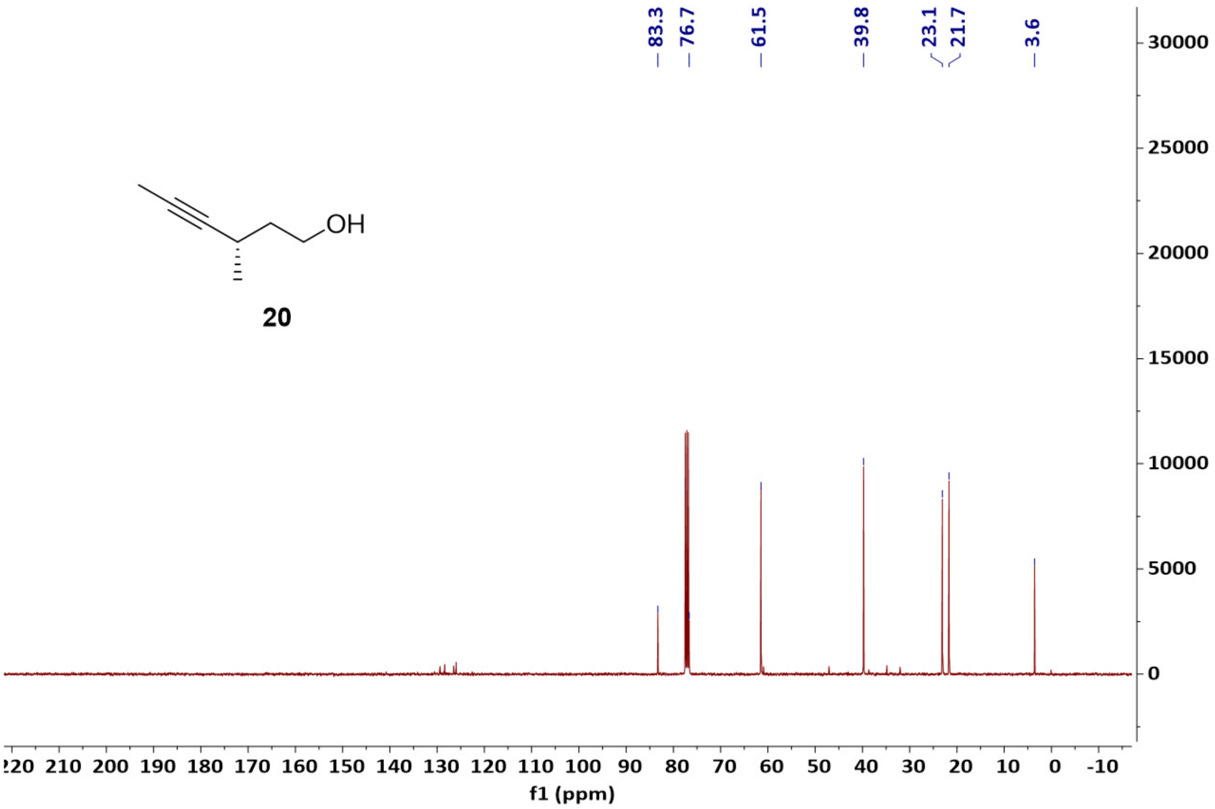

^{1}H NMR (500 MHz, $CDCl_3$)

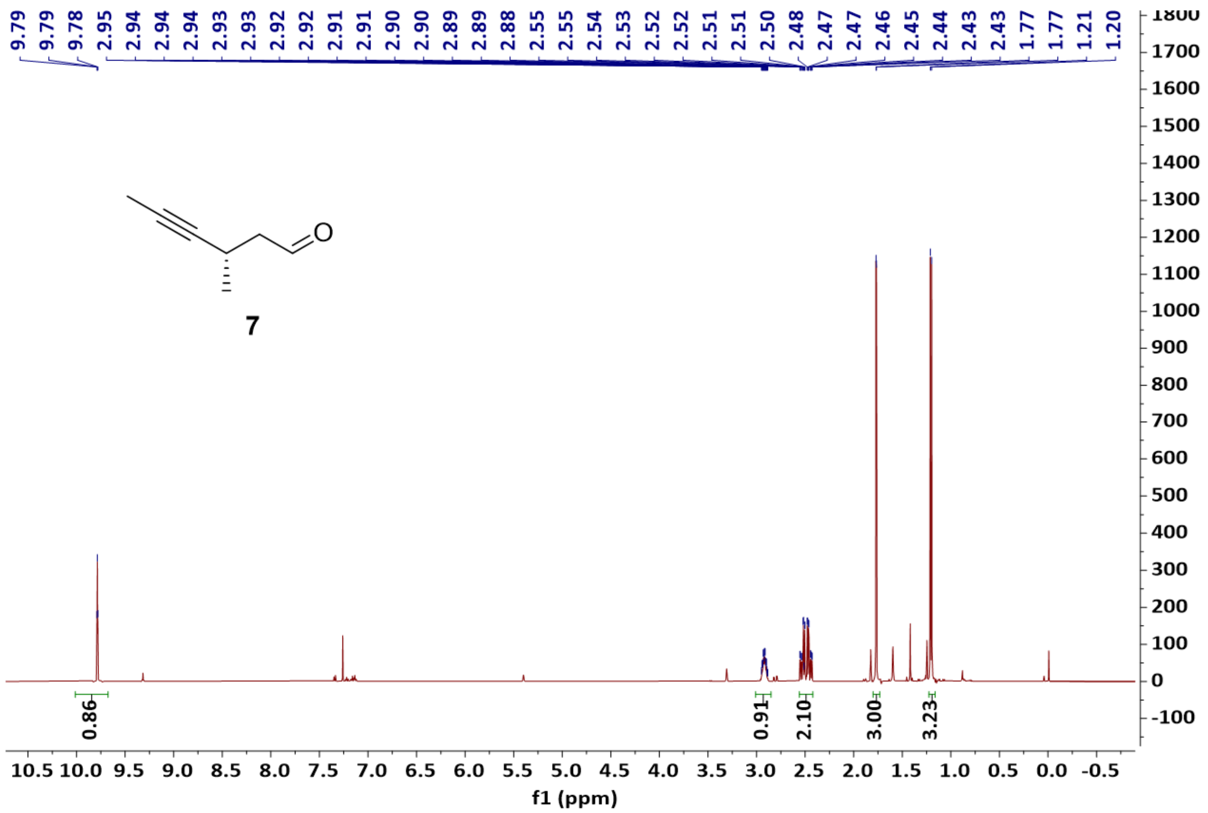

^{13}C NMR (101 MHz, $CDCl_3$)

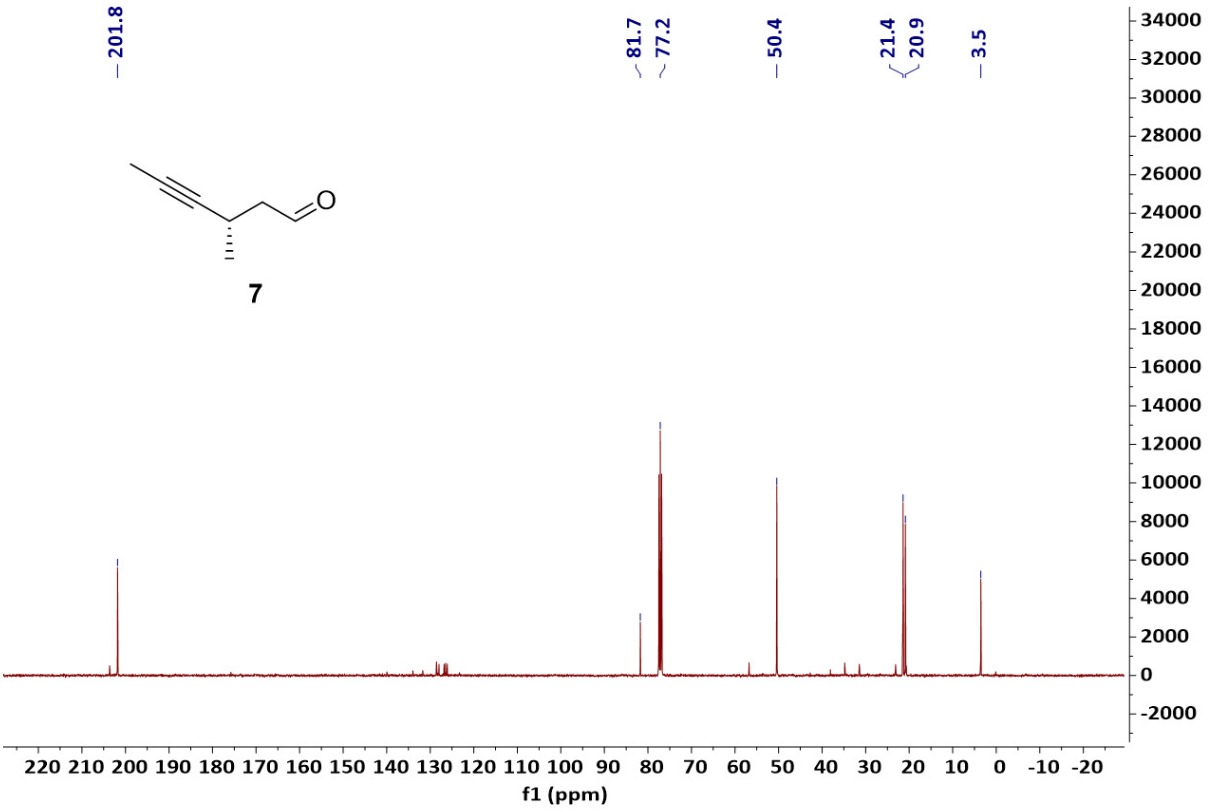

^{1}H NMR (500 MHz, $CDCl_3$)

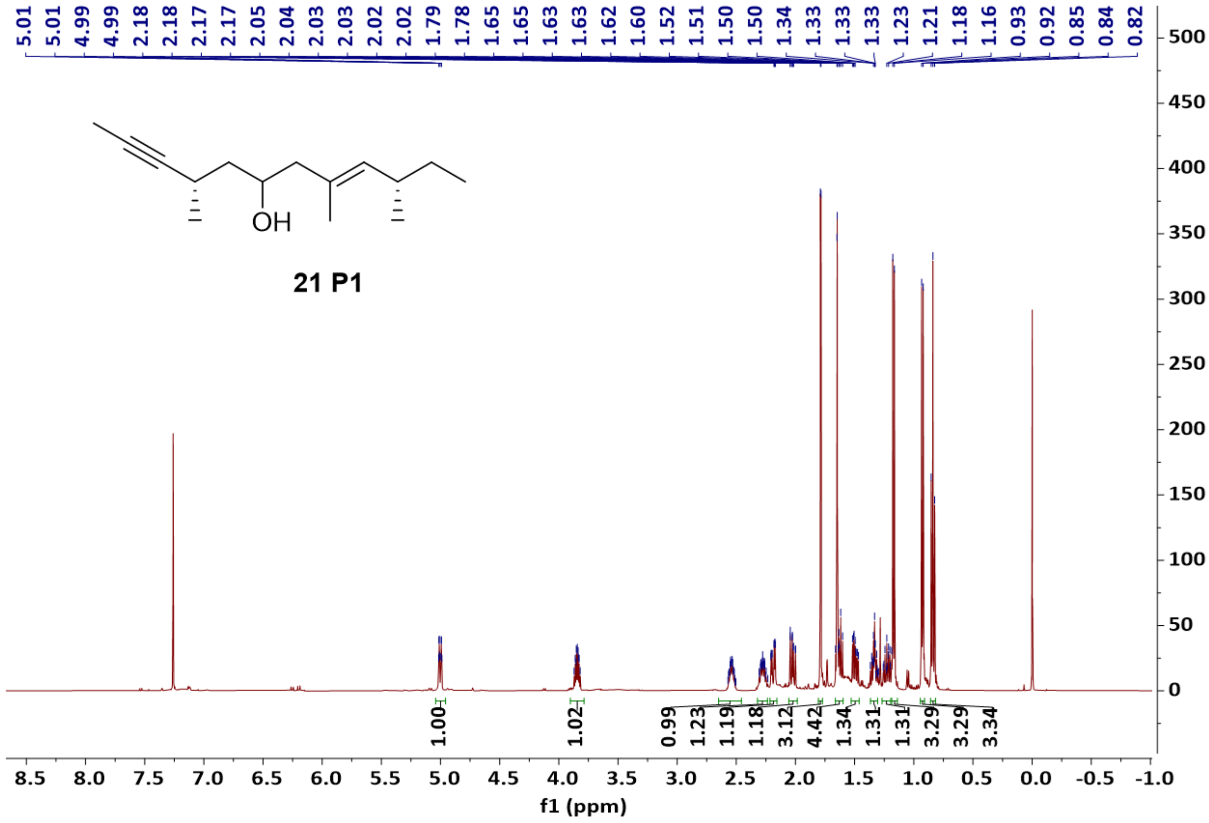

^{13}C NMR (101 MHz, $CDCl_3$)

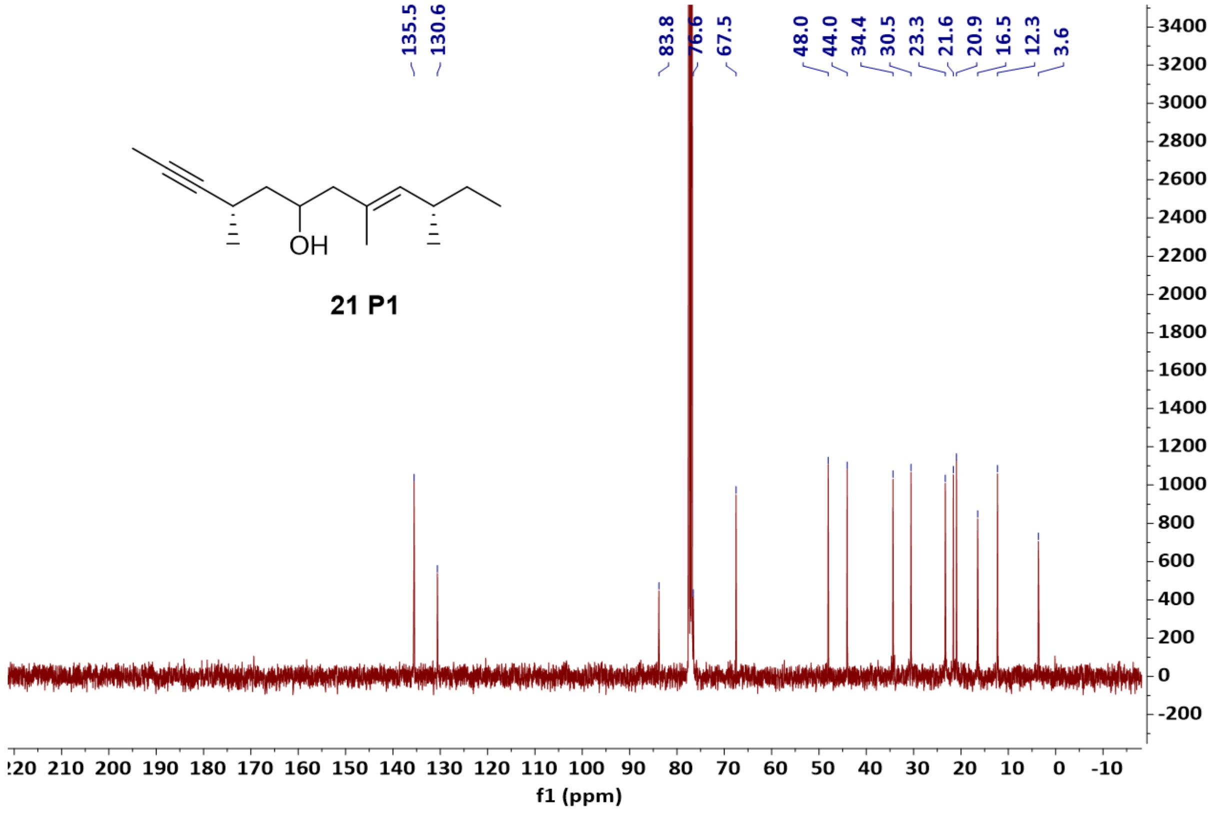

^{1}H NMR (500 MHz, $CDCl_3$)

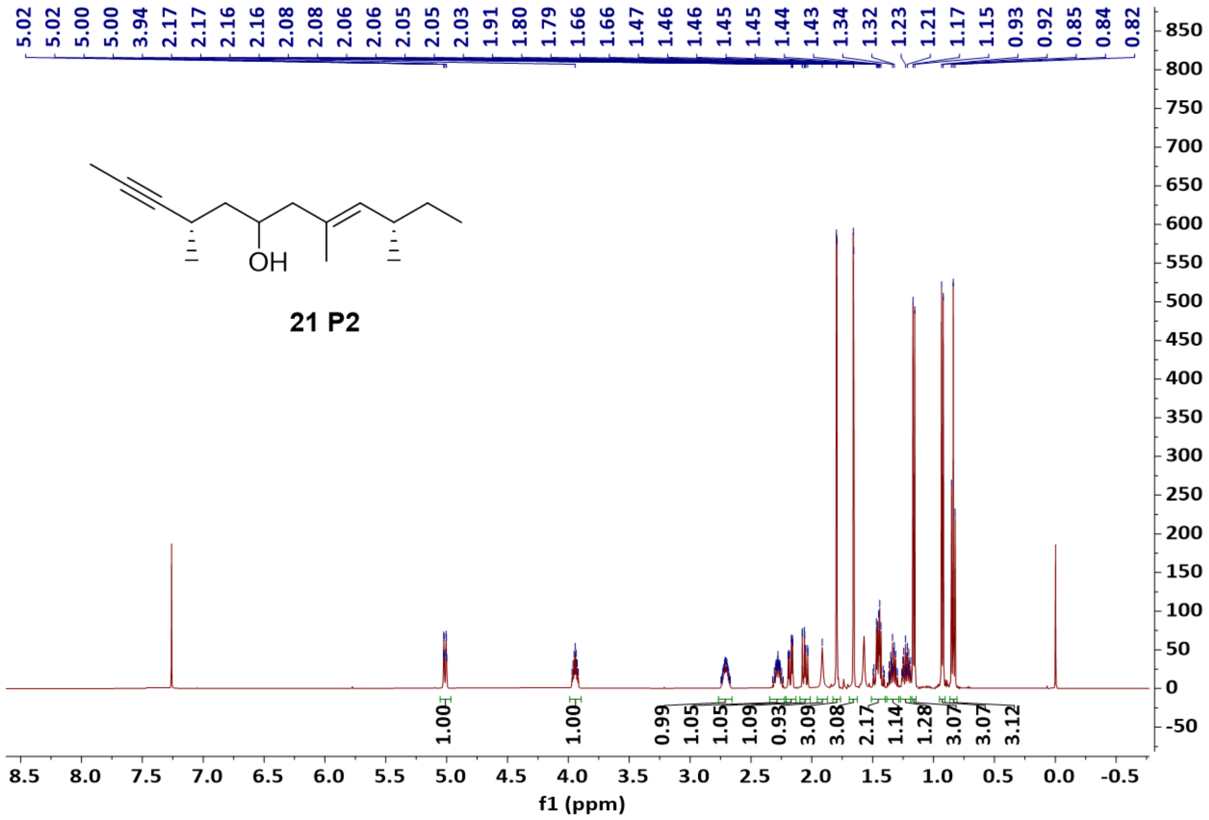

^{13}C NMR (101 MHz, $CDCl_3$)

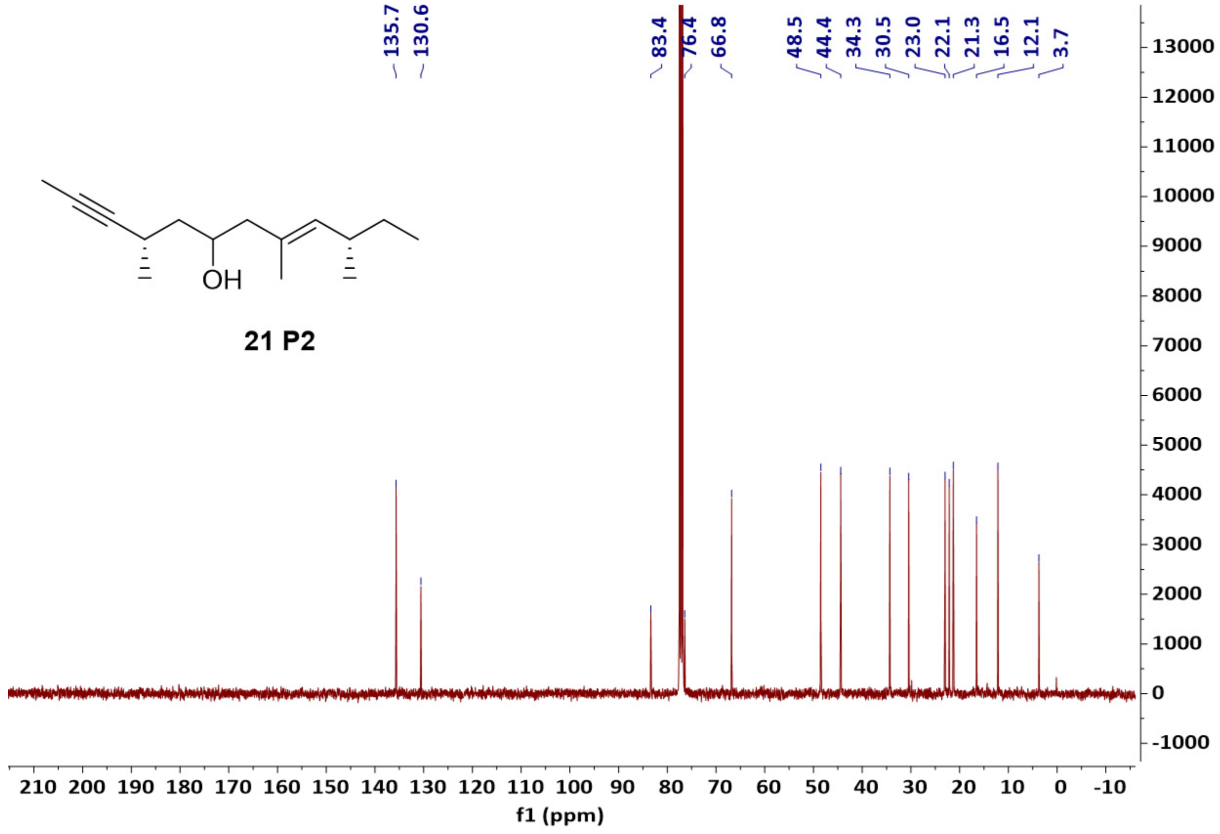

^{1}H NMR (400 MHz, $CDCl_3$)

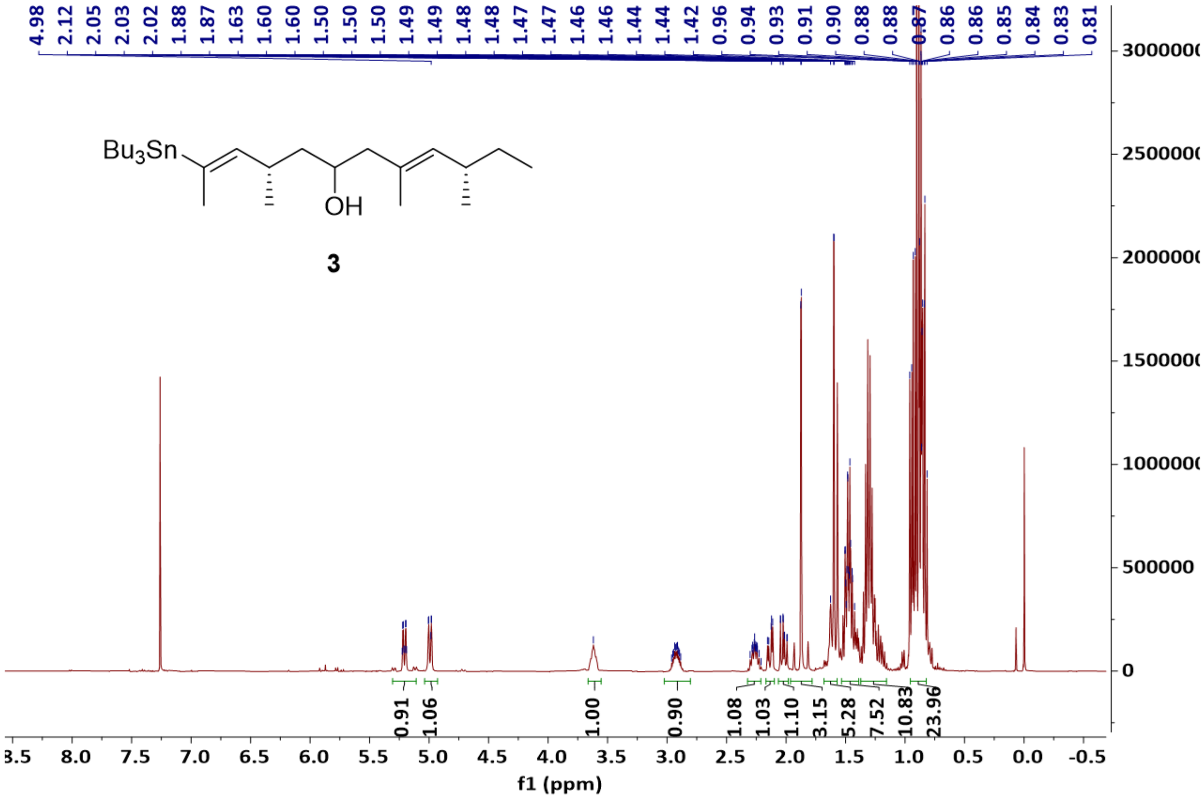

^{13}C NMR (101 MHz, $CDCl_3$)

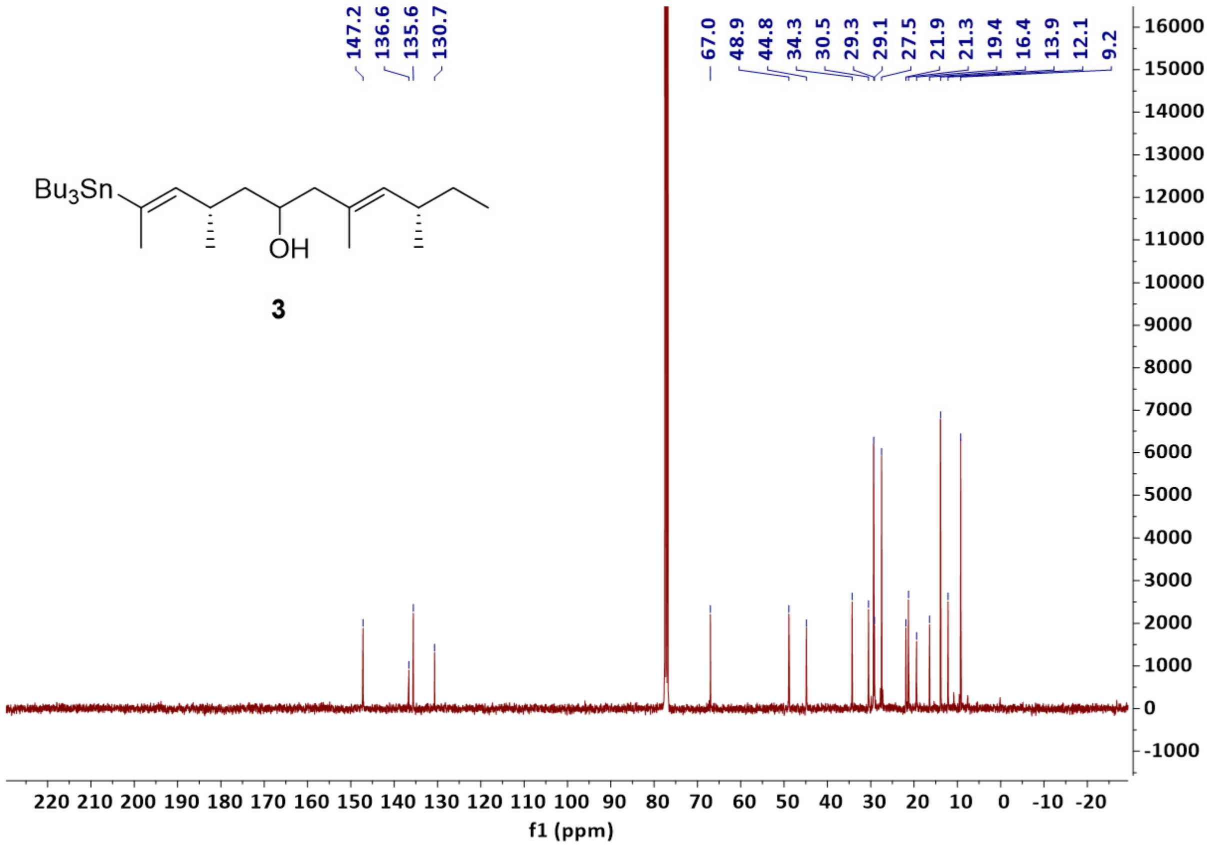

^{1}H NMR (400 MHz, $CDCl_3$)

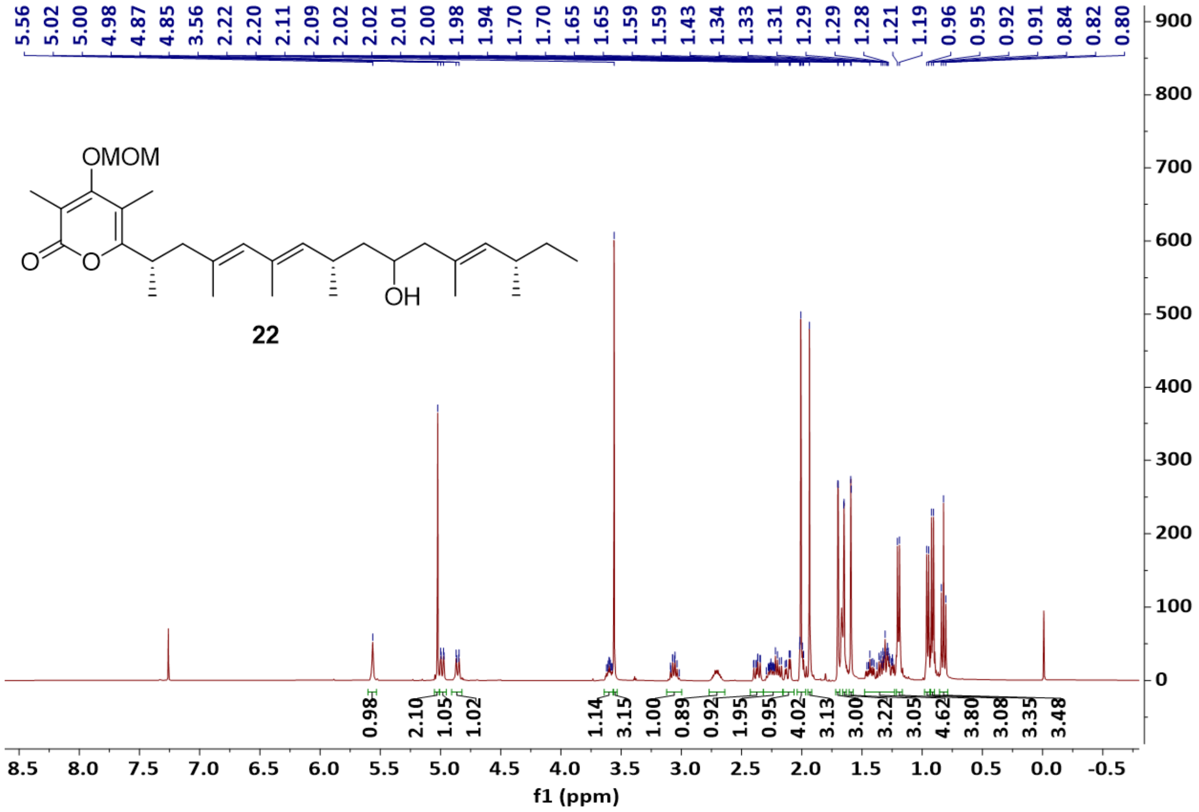

^{13}C NMR (101 MHz, $CDCl_3$)

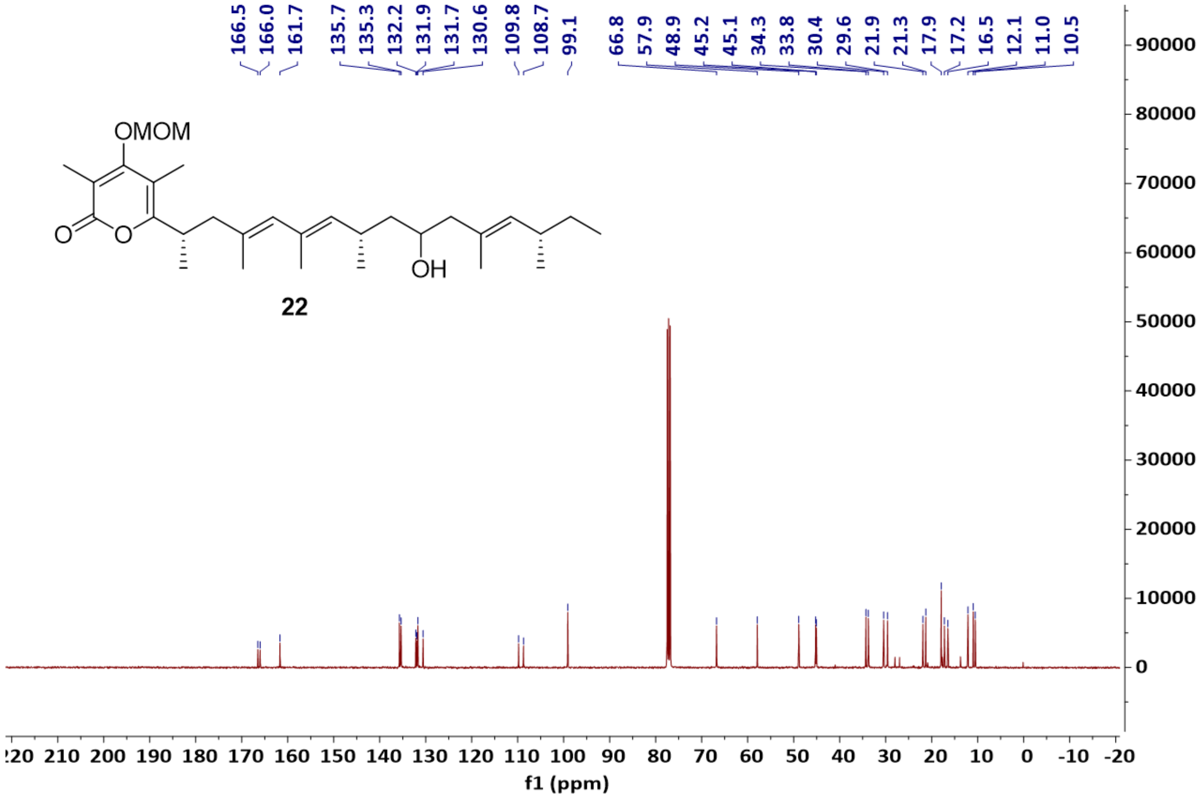

^{1}H NMR (400 MHz, $CDCl_3$)

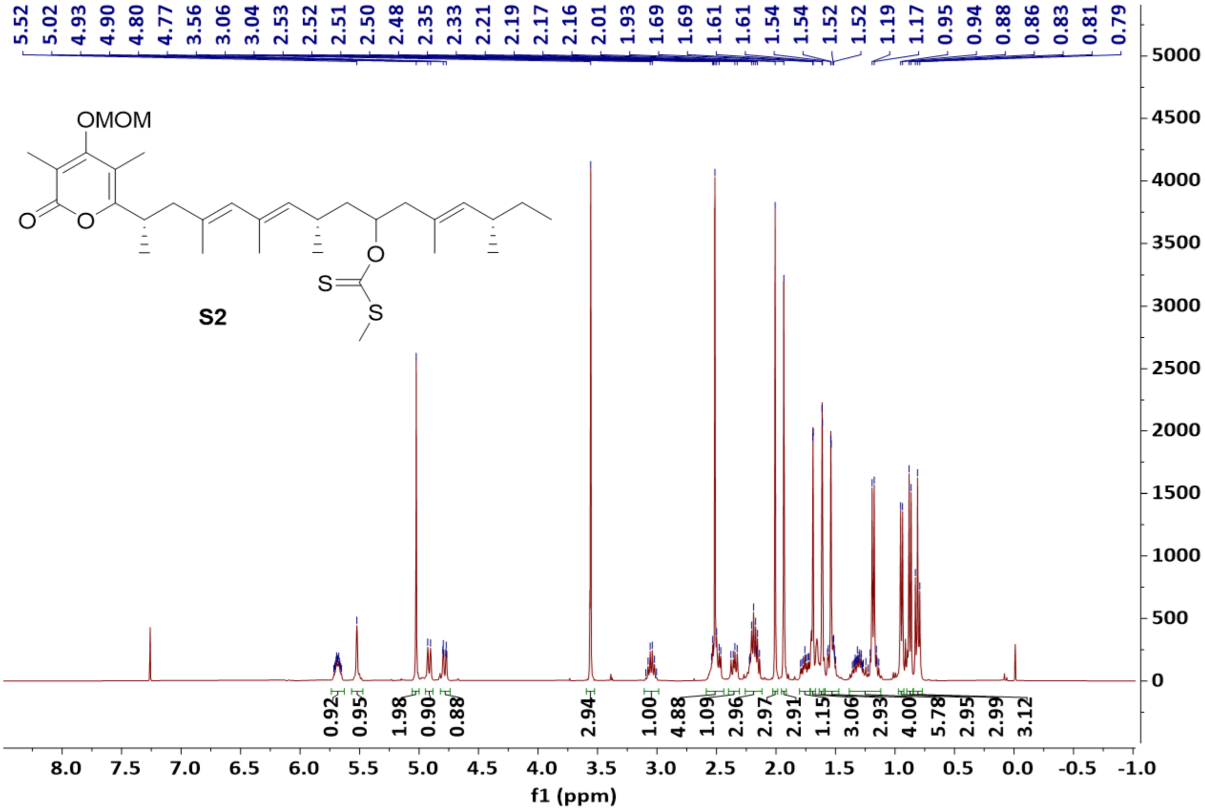

^{13}C NMR (101 MHz, $CDCl_3$)

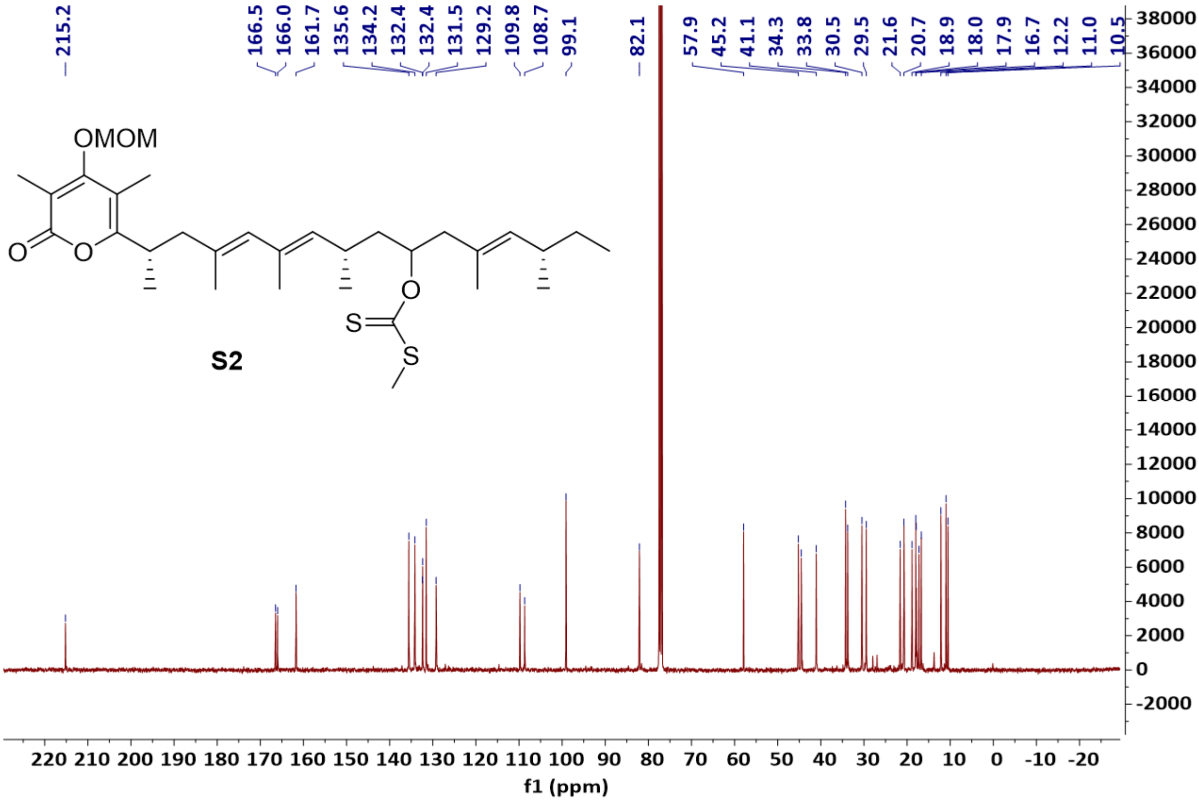

^{1}H NMR (400 MHz, $CDCl_3$)

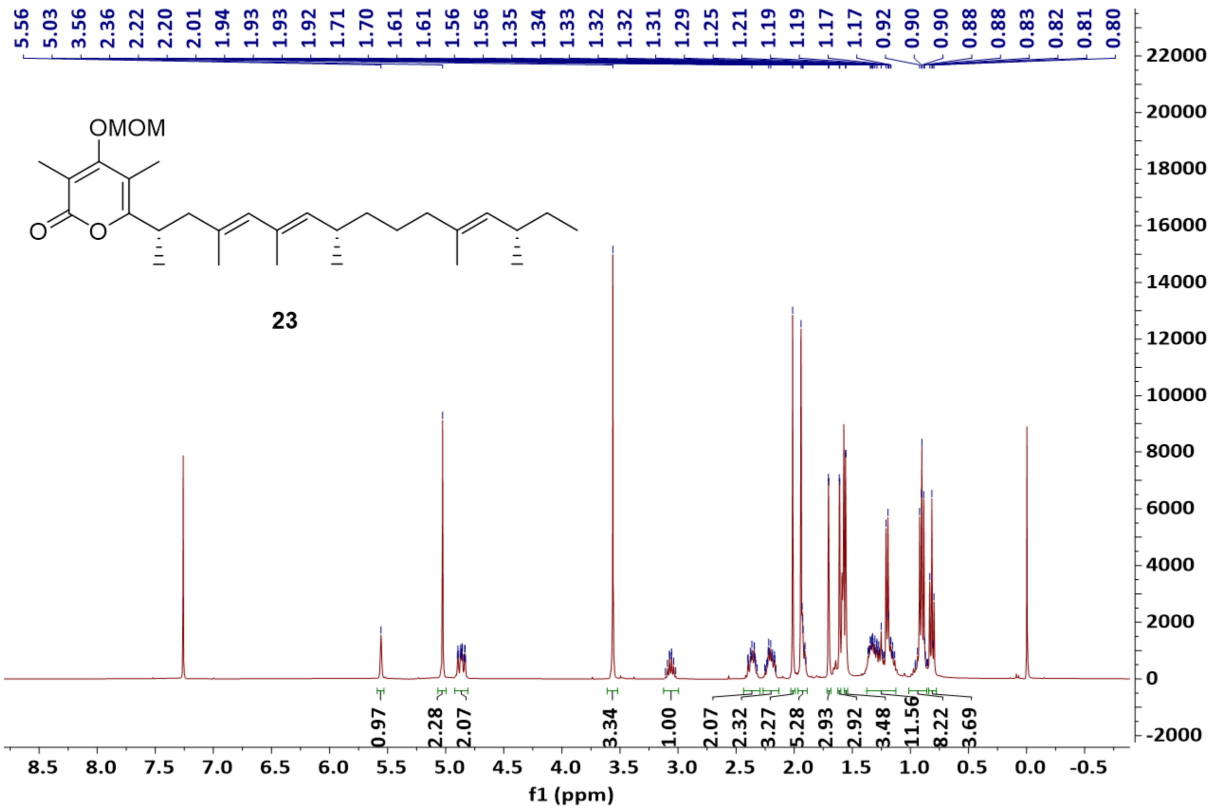

^{13}C NMR (101 MHz, $CDCl_3$)

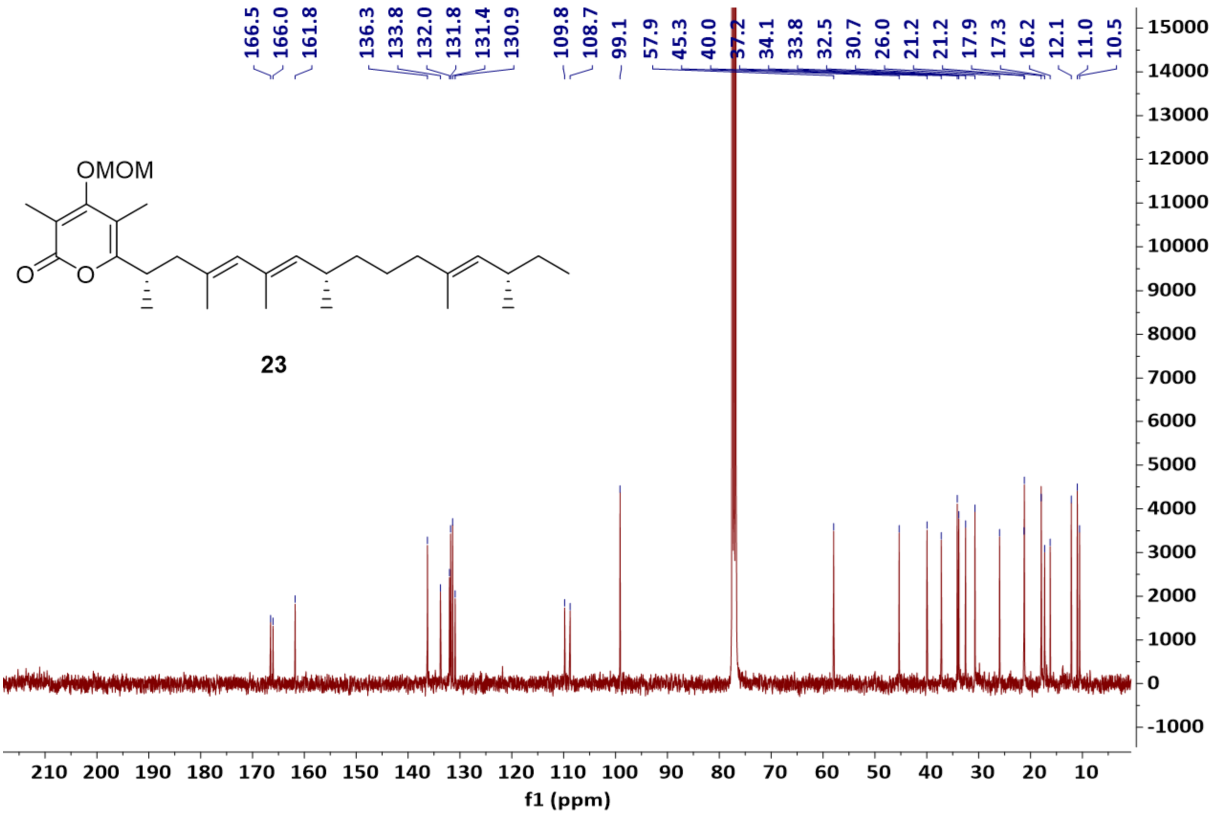

^{1}H NMR (400 MHz, acetone-*d*6)

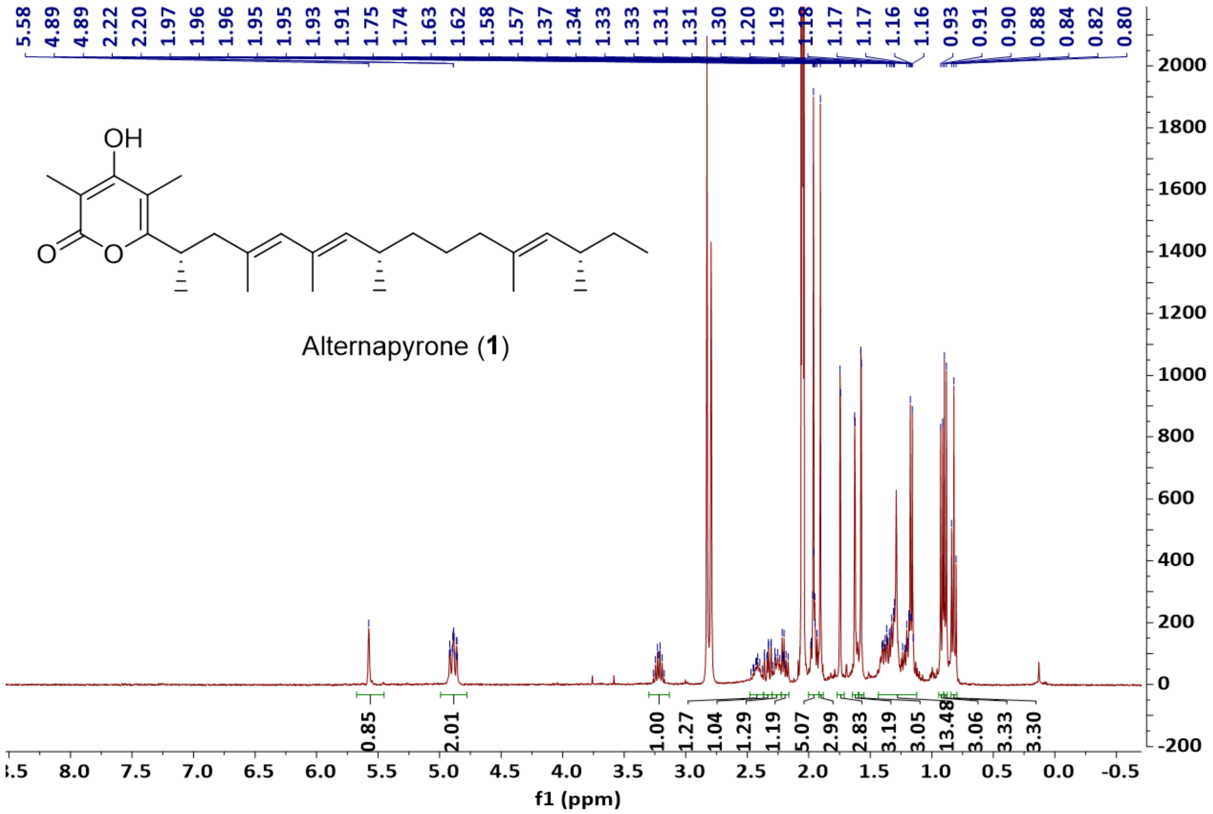

^{13}C NMR (101 MHz, acetone-*d*6)

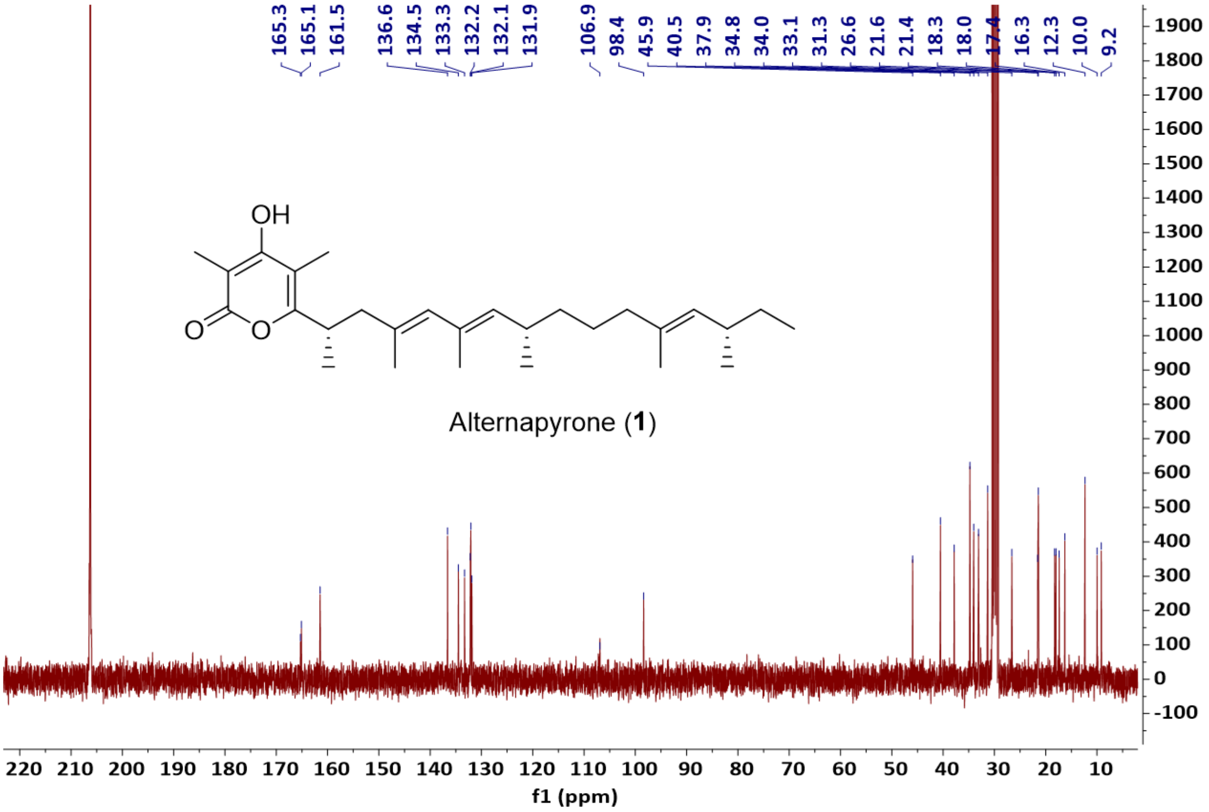

Supplement: Supplementary file 1 [file molecules-30-01597-s001.zip › molecules-3463585-supplementary.pdf]
